# Indocyanine green angiography processing and analysis pipeline for the assessment of indeterminate burn wounds

**DOI:** 10.1117/1.JBO.30.6.065002

**Published:** 2025-06-23

**Authors:** Héctor A. García, Mary I. Junak, Bailey Donahue, Aiping Liu, Adam Uselmann, Brian W. Pogue, Angela L. F. Gibson

**Affiliations:** a University of Wisconsin-Madison, Department of Medical Physics, Madison, Wisconsin, United States; b CIFICEN (UNCPBA – CICPBA – CONICET), Tandil, Argentina; c University of Wisconsin School of Medicine and Public Health, Department of Surgery, Madison, Wisconsin, United States; d OnLume Inc., Madison, Wisconsin, United States; e Dartmouth College, Thayer School of Engineering, Hanover, New Hampshire, United States

**Keywords:** indocyanine green angiography, near-infrared spectroscopy, fluorescence-guided surgery, fluorescence imaging

## Abstract

**Significance:**

Determining the depth of injury in burn wounds is critical to inform surgical decision-making and enhance outcomes. Clinical assessment yields poor accuracy in the early post-burn period, and histologic analysis of biopsies (the gold standard) is time-consuming and clinically unfeasible. Indocyanine green angiography (ICGA) has provided very promising results; however, the evidence is still limited, and the details on instrumentation, measurement setup, and data processing/analysis (when reported) are considerably heterogeneous.

**Aim:**

A processing and analysis pipeline was developed to interpret ICGA data from experimental burn studies in a way that provides objective, generalizable, and reproducible interpretation.

**Approach:**

Different burns were created on the dorsal aspect of adult pigs, and ICGA was performed. ICGA measurements were then compared with different processing steps. Features were extracted from the indocyanine green angiography (ICG) kinetics curves at specific regions of interests and ran individual and group analyses to decide on the wound severity. To this end, the features were analyzed both separately and groupwise.

**Results:**

The repeatability of the study was enhanced by processing steps where ICG curves were normalized by their area under the curve (AUC). Peak value ($I_{\mathrm{MAX}}$), residual AUC (rAUC), mean transit time (MTT), full width at half maximum (FWHM), and ingress ($s_{1}$) and egress ($s_{2}$) slopes presented the strongest correlation with burn severity. MTT and FWHM were almost independent of the processing steps included in the pipeline, providing high reliability between imaging sessions and inter-subject comparisons. Superficial burns presented significantly higher $I_{\mathrm{MAX}}$, rAUC, $s_{1}$, and $s_{2}$, as well as lower FWHM, when compared with the ICG kinetics from normal tissue, whereas the contrary happens for deep burns.

**Conclusions:**

We highlight the utility of a pre-processing step and judicious choice of parameters to use when interpreting ICGA data from indeterminate depth burn wounds to maximize the accuracy in severity estimation.

## 1 Introduction

Indeterminate burn wounds are defined as partial-thickness burns in which the depth of dermal involvement cannot be clearly identified by clinical assessment.^1^^–^^3^ This represents a clinical challenge for burn surgeons because a reliable diagnosis of burn depth is crucial not only for the patient’s recovery but also in terms of medical costs.

**Fig. 1:**
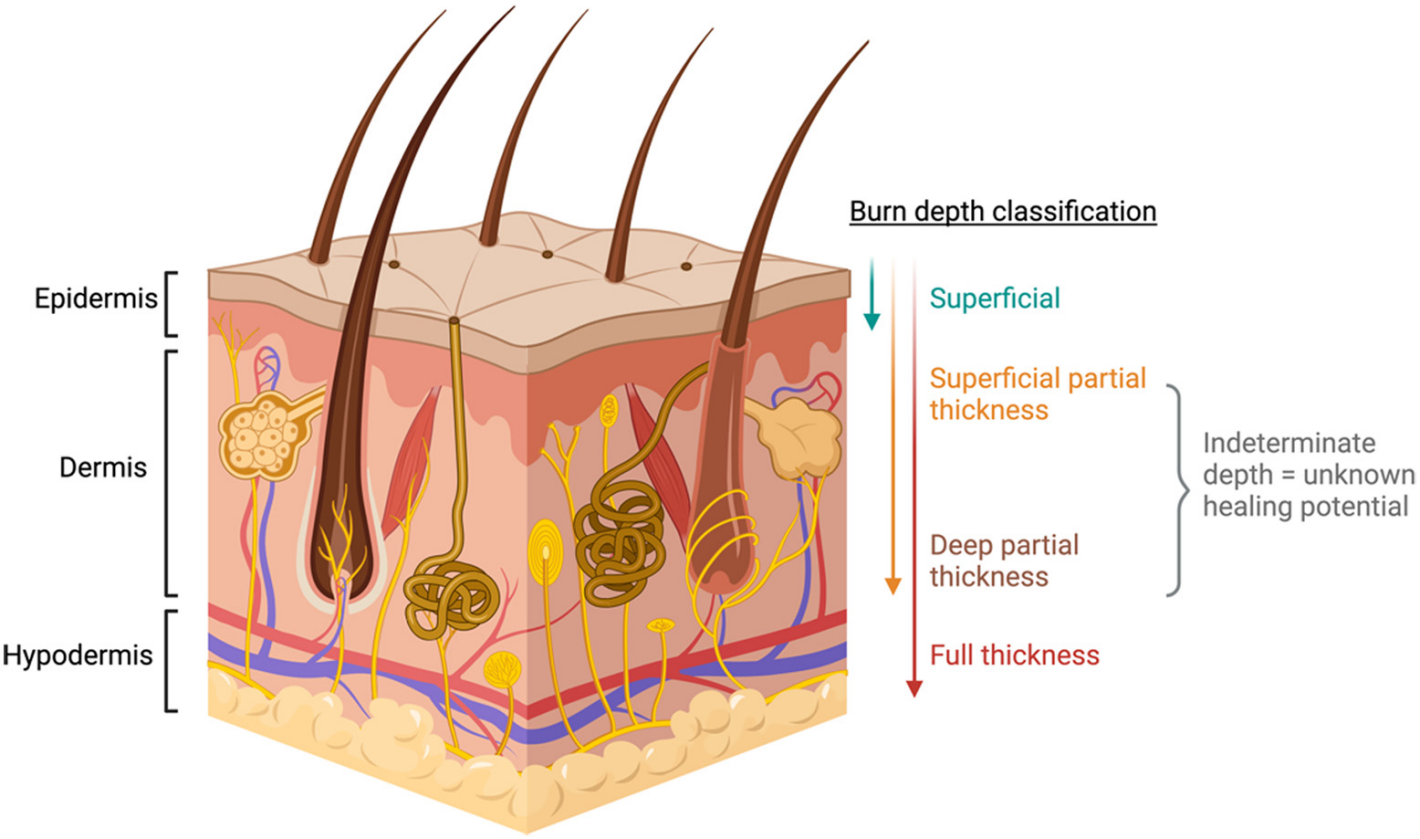
Simplified scheme of the skin layered structure (epidermis, dermis, and hypodermis), together with the burn depth classification according to the type of tissue involved.^4^

Burns are classified into superficial, partial-thickness, and full-thickness burns (Fig. 1).^5^ Although full-thickness burns require excision and grafting for wound closure, and superficial partial-thickness burns usually heal with local wound care alone, management of deeper partial-thickness burns can present a diagnostic dilemma. Shortly after injury, it is difficult to determine the healing potential of intermediate-depth burns based on visual assessment. Inaccurate assessments of burn depth can lead to unnecessary surgical intervention with over-excision of regenerative tissue and creation of potentially unwarranted donor sites.^5^^–^^7^ Relying solely on clinical assessment to decide on the severity of indeterminate burn wounds is risky because the accuracy of this approach ranges from 50% to 75%.^6^ On the contrary, histological analysis of biopsies is considered the gold standard, but it is invasive, painful, limited to very small regions, and can require a clinically undesirable length of time for tissue fixation and pathological report.^6^^,^^7^

Several different technologies have been proposed to overcome these issues. Among them are nuclear medicine imaging^8^ and thermography,^9^ of which both have been concluded less reliable. More recently, several optics-based techniques have been tested, such as laser speckle imaging, hyperspectral imaging, optical coherence tomography, and photoacoustic imaging, with each of them having different pros and cons as well as inconclusive degrees of success as of yet.^10^

Indocyanine green angiography (ICGA) is an optical imaging approach,^5^ which consists of illuminating the damaged tissue at an appropriate wavelength—typically in the near-infrared (NIR) region of the electromagnetic spectrum—and recording the NIR fluorescence signal generated by the excited indocyanine green (ICG) molecules that are simultaneously injected into the patient’s circulatory system. This technique has been widely used for several different applications,^11^^–^^14^ including the study of indeterminate burn wounds. ICG is a small molecule dye ($C_{43}H_{47}N_{2}{\mathrm{NaO}}_{6}S_{2}$, relative molecular mass 774.96) that binds to serum proteins and behaves as a macromolecule in circulation.^15^ Because of this, ICG kinetics show notorious differences between healthy and damaged tissues in terms of uptake and clearance,^16^ which can be exploited with suitable imaging techniques and devices allowing in some cases for real-time measurements, assisting physicians and surgeons in applications such as fluorescence-guided surgery (FGS).^17^ However, there are no established protocols for use of the devices, techniques for analysis, and the data processing/analysis required. As the first publication on this topic in 1995 by Sheridan et al.,^18^ less than 10 original papers have been published in the course of 30 years.^6^^,^^19^^–^^24^ In all cases, the reported results were based on analyzing either the raw fluorescence signal acquired with the imaging device or the ratio between the signal from the burn region of interest (ROI) and the signal coming from normal tissue considered as a reference. Computing the ratio of damaged to normal tissue is a common technique to quantify the image, used in several applications of tissue ICGA.^25^^–^^27^ Surprisingly, the exact way in which this ratio is computed is very rarely detailed, and in general, the technique does not fully take advantage of the temporal data which is rich in information about the tissue vascular perfusion.

Technical details such as information about the light sources, imaging capabilities, and working distance (WD) from the camera to the tissue surface are not typically reported, and these can affect the magnitudes of the signals. Sheridan et al.^18^ and Still et al.^20^ provide some basic details on laser power and fluence, whereas the latter mention a WD of 53 cm; in addition, Jiang et al.^24^ claim the WD in their study is kept constant, but no numeric value is informed. Apart from these, none of the authors reflects any concerns about those technical aspects (such as the field of view uniformity or the curvature of the imaged tissue surface) that could possibly introduce artifacts in their studies. Only Wongkietkachorn et al.^6^ makes a brief mention of motion artifacts and the possible consequences of not properly dealing with them, and Jerath et al.^19^ informs of a background subtraction step for their image and processing analysis. Perhaps most problematic is the fact that these systems rarely have a homogeneous field of sensitivity, and so comparisons or ratios estimated from different tissues such as burn and normal tissue can be drastically affected by the instrument’s performance and use.^28^

The specific clinical burn data reported is also highly variable because, in some cases, no information is supplied at all, whereas in others, details are provided of the burn location and etiology, the days post-burn, and even the ROI sizes. Unfortunately, the studies based on human data^6^^,^^18^^,^^20^^–^^22^ often report the least instrumentation and technique details, whereas those based on animal models^19^^,^^23^^,^^24^ often inform much more (providing also details about the methods used for burn generation, size, and amount), given the controlled nature of their studies. The ICG dose administered is always reported, with values ranging from 0.1 to 0.5  mg/kg, but the injection technique (bolus or slow infusion) is solely detailed by Jerath et al.^19^ In addition, in most human studies, the ICGA acquisition time is done at different numbers of days post-burn, up to 5, but it is likely that changes in vascular kinetics occur in this timeframe due to tissue healing or necrosis evolution.^21^^,^^22^ Given the value of clinical data, it would be very desirable to have more robust technique documentation, data collection, and processing information about ICGA in human studies.

In general, ICGA is done with video imaging, recorded or image captured during the first 5 min following the bolus ICG injection. The frames per second (fps) of the videos and the number of images—when detailed—present high variability because these parameters strongly depend on the particular acquisition system used in each study.

The type of analysis across studies is predominantly qualitative, being the peak value of the kinetics curve or of the captured images as the most common marker. The only exception is the study by Jerath et al.,^19^ where a semi-quantitative model linking the fluorescence signal with the burn depth was introduced. Also, Jerath et al., Still et al.,^20^ and Dissanaike et al.^22^ discuss the influence of the optical behavior of tissues (in terms of their light absorption and scattering properties) on the measurements.

Although the goal of most studies is to determine the benefits of using ICGA as an aid to clinical assessment of the severity of burn wounds, the lack of system, technique, and protocol details (and their variability) will limit useful conclusions. Here, the post-imaging processing and analysis steps performed in each study are examined for reproducibility. The goal was to define the steps that should be consistently incorporated into future human study protocols. This work was developed in a swine model where burns of varying depths could be systematically created for ICGA measurement to extract ICG kinetics curves from a number of ROIs. The information from those curves was correlated with the time of burn duration, as an indicator of damage severity.

This paper is structured as follows. Section 2 briefly depicts the general background of ICGA applied to assessing indeterminate burn depths; Sec. 3 is dedicated to the description of the proposed processing and analysis pipeline; Sec. 4 presents the details on the swine study protocol as well as on the imaging device and the experimental setup; Sec. 5 shows the results the effect of applying different processing steps on the acquired data and also their effect on the individual as well as on the group analysis; Sec. 6 is centered on discussing the results; and finally, Sec. 7 is focused on the main conclusions of this work.

## 2 Principles of ICGA

**Fig. 2:**
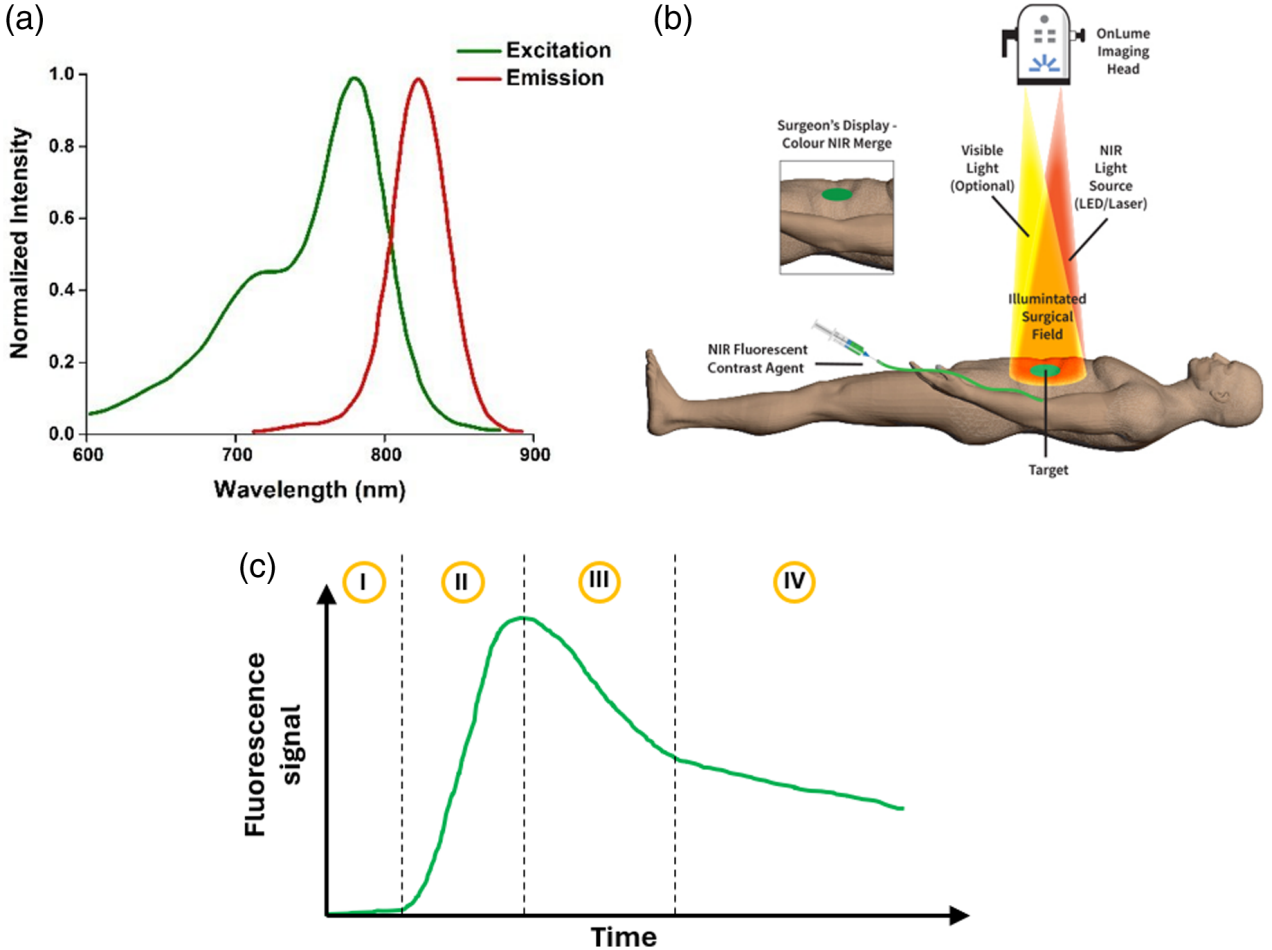
(a) ICG excitation and emission spectra (taken and adapted from Ref. 30). (b) Scheme of a typical ICGA measurement (taken from Ref. 31). (c) Typical ICGA kinetics curve.

Indocyanine green is one of the few Food and Drug Administration–approved medical dyes used as an extrinsic fluorescence contrast agent for FGS.^29^ It absorbs NIR light at a peak around 800 nm and presents a maximum emission peak at 830 nm [Fig. 2(a)], a range of wavelengths where biological tissues present relatively low absorption and, consequently, photons can penetrate living tissue up to a few centimeters.^32^^–^^34^ In the context of an ICG angiography measurement, suitable NIR light sources [such as light-emitting diodes (LEDs) or lasers] illuminate the tissue once the ICG bolus has been injected into the patient. This light is absorbed by the fluorophore molecules and reemitted as photons of larger wavelengths. Due to the isotropic nature of the fluorescent emission and the highly scattering characteristics of biological tissue, a great proportion of light is diffusely reflected back to the tissue surface, which allows for the detection of the fluorescent signal under a combination of a recording camera and suitable optical filters [Fig. 2(b)]. A typical ICG kinetics curve, i.e., the time evolution of the detected fluorescence signal, is shown in Fig. 2(c). In general, this curve can be split into four distinct moments: (I) an initial background signal that arises from starting the recording at the moment when the ICG is injected and which very rarely provides useful information, (II) a rapid increase of the signal from the background level up to the peak value, (III) a fast decrease indicating the start of the ICG washout process, and (IV) a decrease continuing the washout process but at a much slower rate. The second moment is associated with the inflow of ICG, whereas the last two moments are associated with its outflow and can be explained by considering the mechanisms of perfusion and permeability.^35^

The hypothesis on which many studies are based is that the ICG kinetics curves for healthy and diseased tissues are noticeably different because the latter show perfusion and permeability issues (not present in normal tissue) that have a direct and strong impact on the shape of the kinetics curve.^5^^,^^35^

## 3 Proposed Processing and Analysis Pipeline

**Fig. 3:**
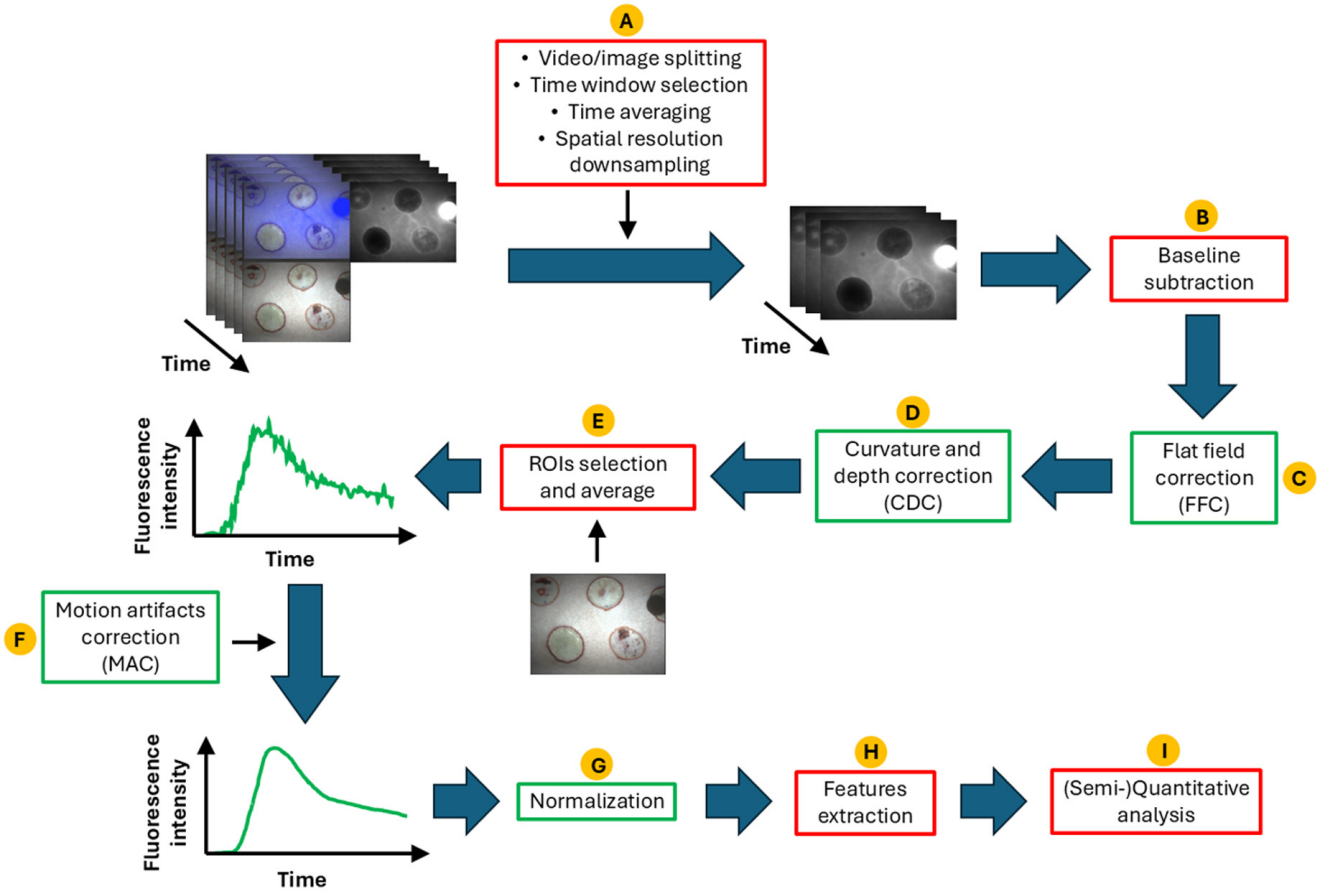
Diagram of the pipeline needed to perform reliable data processing and analysis. The red boxes represent mandatory steps, whereas the green ones may be omitted due to user preferences or device limitations.

In this section, we focus on the necessary data processing and analysis strategies to robustly assess the severity of indeterminate burn wounds. This does not mean that details such as the hardware selection, the choice of a particular measurement setup, and the image or video acquisition process are unimportant; on the contrary, the end user must pay attention to those details and apply the processing and analysis pipeline that best adapt to the specific conditions of their study. We recommend following the workflow proposed by Noltes et al.^14^ to account for those details before proceeding with the data processing and analysis. The pipeline proposed here and depicted in Fig. 3 is based on the principle that the acquired data cannot be directly used in its fully raw status to proceed with interpretation, but rather it requires a minimum level of processing. However, too much processing may corrupt the outcomes and lead to biased conclusions, so a balance must be found. The discussion at the end of this paper reflects the astounding heterogeneity and lack of details in the published literature that led to the need for this suggested pipeline. The diagram shown in Fig. 3 consists of a series of red and green boxes representing mandatory and optional steps, respectively. These are described in Secs. 3.1–3.9.

### 3.1 Data Selection (A)

This step is considered mandatory because, in general, ICGA datasets consist of ultra-high-definition (UHD) video or image files that can easily reach sizes of hundreds of megabytes or even several gigabytes. Moving further into the pipeline without discarding the unnecessary information would imply extremely long computation times and/or excessive storage capabilities. The way in which the irrelevant data are discarded relies on the particular way in which the chosen acquisition system exports the measured data. For instance, some devices store video or image files where the complete frame is divided into a white light field, a fluorescence field, and the overlay of both,^31^ in which case a splitting step is necessary. The time window selection is needed to help the processing pipeline focus on the important part of the data. The time averaging refers to the option of reducing the sampling rate of the acquisition but at the same time increasing the signal-to-noise ratio of each frame. The spatial resolution downsampling can greatly speed up the time computations, especially when UHD is not really needed.

### 3.2 Baseline Subtraction (B)

The process of baseline subtraction is meant to remove the influence of the background noise on the overall fluorescence signal. In general, this is done by selecting a few frames at the start of the experiment to compute an average which is then subtracted from the full recording.

### 3.3 Flat Field Correction (C)

The necessity of this step will depend on the system’s characteristics and the user’s criterion regarding the area to be imaged. If the device presents a uniform NIR illumination, this step can be omitted; if not, it is highly recommended to focus the study on the region of the field of view (FOV) where the illumination is maximized (which generally occurs at the center). If this is not possible, a flat field image ($I_{F}$) and a background image ($I_{B}$) can be used to correct a raw image ($I_{R}$) to obtain a corrected image ($I_{C}$) as follows^36^^,^^37^:

$$I_{C}=\frac{I_{R}-I_{F}}{I_{F}-I_{B}}M_{FB},$$ (1)

where $M_{FB}$ is the mean value of the difference in the denominator of Eq. (1). The image $I_{F}$ is intended to show the intensity distribution over the imaged plane, and as such, it can be obtained by imaging a flat surface with a homogeneous fluorophore distribution. The image $I_{B}$ records the dark counts acquired by the camera when the device’s light sources are off, providing information about the influence of ambient light on the measurements. It is worth mentioning that applying a flat field correction (FFC) will not lead to the same exact results as using a device with a uniform illumination field and/or measuring at the center of the FOV because the output will suffer from some degradation. Therefore, the reader is strongly encouraged to carefully choose the measurement setup to leave this correction as the final strategy only if there is no alternative.

### 3.4 Curvature and Depth Correction (D)

**Fig. 4:**
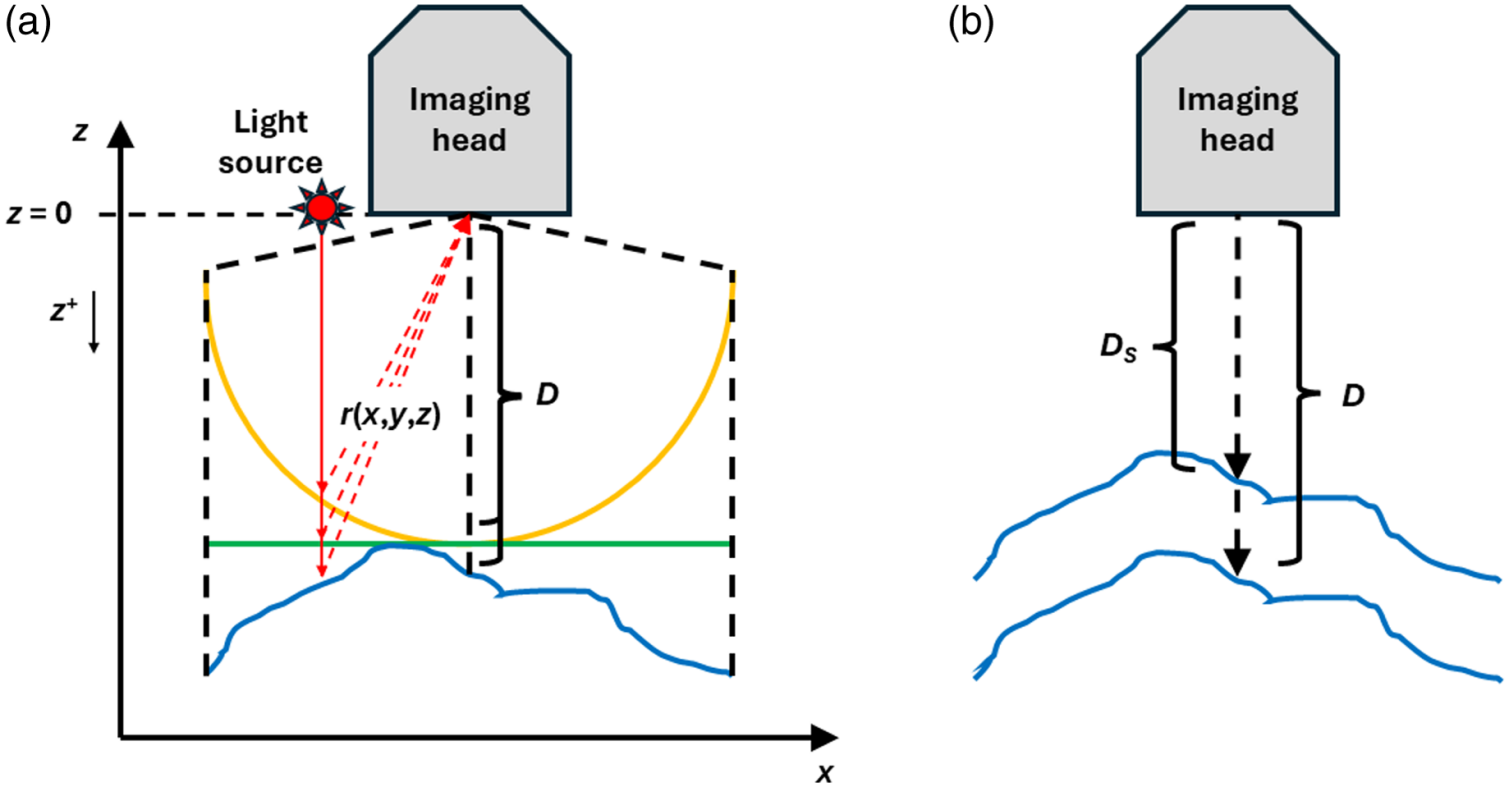
(a) Differences in illumination and imaging arising from tissue curvature and distance to the camera. (b) Correcting data acquisition for different working distances D and $D_{S}$.

Assuming that all points in the tissue surface emit fluorescent light isotropically, the intensity I(x,y,z) detected by the imaging device will depend on the distance r(x,y,z) to the camera according to the relation

$$I(x,y,z)=\frac{P(x,y,z)}{4\pi r^{2}(x,y,z)},$$ (2)

where P(x,y,z) is the power (energy per unit of time) of the pixel at the set of coordinates (x,y,z) [although the recorded two-dimensional (2D) image carries explicit information only from the x to y plane, the distance from the tissue to the camera affects the detected intensity, and this is implicitly related to the z coordinate, which needs then to be taken into account in Eq. (2)]. If the surface to be imaged was a perfect concave spherical shell with a constant distance to the center of the camera [Fig. 4(a), orange line], the distance r would just match the working distance D (i.e., r(x,y,z)=D), and I(x,y,z) would not depend on this distance, meaning that differences in pixel intensities would solely reveal the differences in tissue kinetics. In reality, this situation is unlikely because the tissue to be imaged generally presents curved and irregular surfaces away from the camera in a “convex-like” fashion [Fig. 4(a), blue line], implying that the differences in the detected intensity will depend on the kinetics and the distance of each point to the camera. Moreover, the detected images or frames, which are 2D arrays of intensity values, can be processed by accounting only for the information given by the distance of each pixel to the center of the camera; however, no information can be retrieved regarding the z coordinate. In the best case, these irregularities are so small that the surface might be considered flat [Fig. 4(a), green line], yet the distance from each point to the camera plays an important role. Nevertheless, in this particular case, Eq. (2) can be corrected in a more predictable way by setting $r(x,y,z)=\sqrt{D^{2}+x^{2}+y^{2}}$ instead of r(x,y,z)=D.

It should be noted that if measurement sessions are performed with different working distances D, a direct comparison among sessions is impossible, and a correction must be applied to make the datasets comparable. To this end, it is possible to make use of Eq. (2) to refer all the measurements to one single standard working distance $D_{S}$ [Fig. 4(b)], leading to a corrected image $I_{D_{s}}$ of the form

$$I_{D_{s}}(x,y,z)=I_{D}(x,y,z)\frac{D^{2}+x^{2}+y^{2}}{D_{s}^{2}+x^{2}+y^{2}},$$ (3)

being $I_{D}(x,y,z)$ the non-corrected data obtained at the working distance D. Of course, instead of doing this, it is preferable to always measure at the same WD to avoid unnecessary data manipulation.

### 3.5 ROIs Selection and Averaging (E)

Once the 2D images or video frames have been properly loaded, adapted, and corrected, the ROIs to process and analyze the data must be chosen. To this end, the use of white field images is highly recommended, as suggested in Fig. 3. Ideally, the ROIs should be placed as close to the center of the FOV as possible to minimize the pitfalls mentioned above, whereas some or all the correction strategies introduced so far could be applied. Then, a simple mathematical average over all the pixels inside each ROI will produce kinetic curves such as the one depicted in Fig. 2(c). Any kinetic curve calculated in this way can be interpreted as the mean behavior of the signal for the selected ROI.

In general, tissue kinetics (either normal or damaged) are highly heterogeneous in nature and individual kinetics phenomena may be lost during the averaging process if ROIs are too large. Alternatively, extremely small ROIs would give rise to unnecessarily variable kinetics curves (again due to the great heterogeneity), making any comparison among ROIs challenging. Hence, when selecting ROIs, it is recommended to find a balance between these two extremes. In addition, it is desirable to avoid including clearly and visibly distinguishable tissue regions inside the same ROI.

### 3.6 Motion Artifacts Correction (F)

**Fig. 5:**
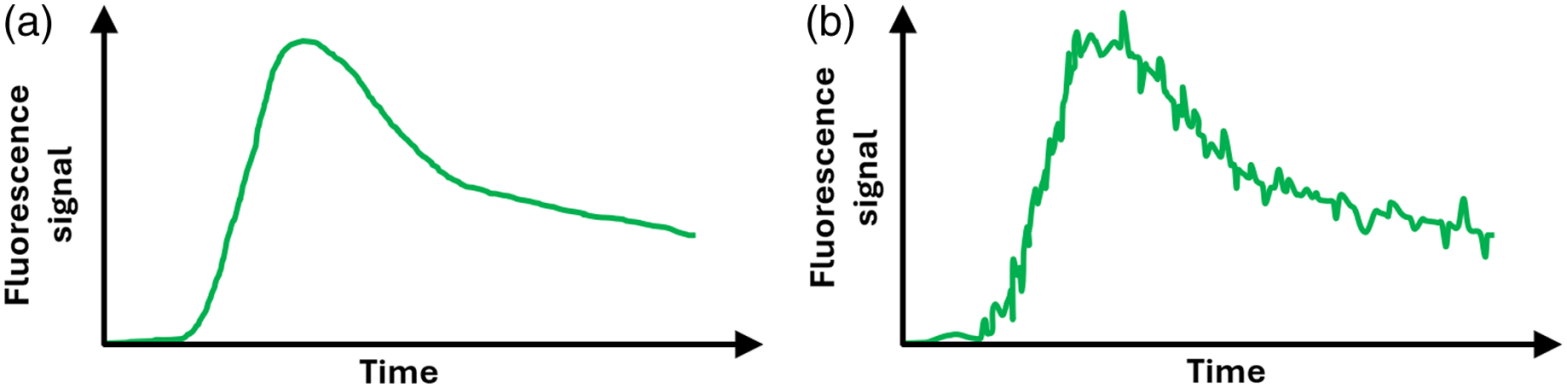
(a) Signal without motion artifacts. (b) Signal with motion artifacts.

When no motion artifacts are present, the average ROI kinetics curve shows a smooth behavior over time, as schematized in Fig. 5(a). If present, motion artifacts will introduce undesired spikes and high-frequency oscillations in the signal [Fig. 5(b)], which may hinder the identification of features such as the start of the uptake or the position of the peak value, among others. Several different strategies exist for dealing with motion artifacts if physical or chemical restraint of the subject is not feasible. All the computational methods developed for this purpose are based on separating those parts of the signal attributed to an undesired motion, from those parts that behave as expected. In the case of ICG kinetics in burn wounds, this task is relatively easy because the typical times in which the signal changes are of the order of a few seconds [thus generating the smooth behavior seen in Fig. 5(a)], whereas the presence of motion artifacts introduces the much faster spikes and oscillations described above. The identification of motion artifacts can be done manually (the user selects the artifacts in the signal one by one) or programmatically (a software or a script makes use of a collection of criteria to extract the artifacts).

Once the motion artifacts have been identified, the last step is to correct them. Among the several options available, the simplest one is to remove the data points in which the artifacts are present; another possibility is to smooth either the whole dataset or just the affected portion of the signal; a third strategy is to use principal component analysis filtering to remove undesired components of the signal.^38^ Many other tools exist that will depend on the type of study being performed, the quality of the acquired data, and the specific goals to be achieved by the study.

### 3.7 Normalization (G)

**Fig. 6:**
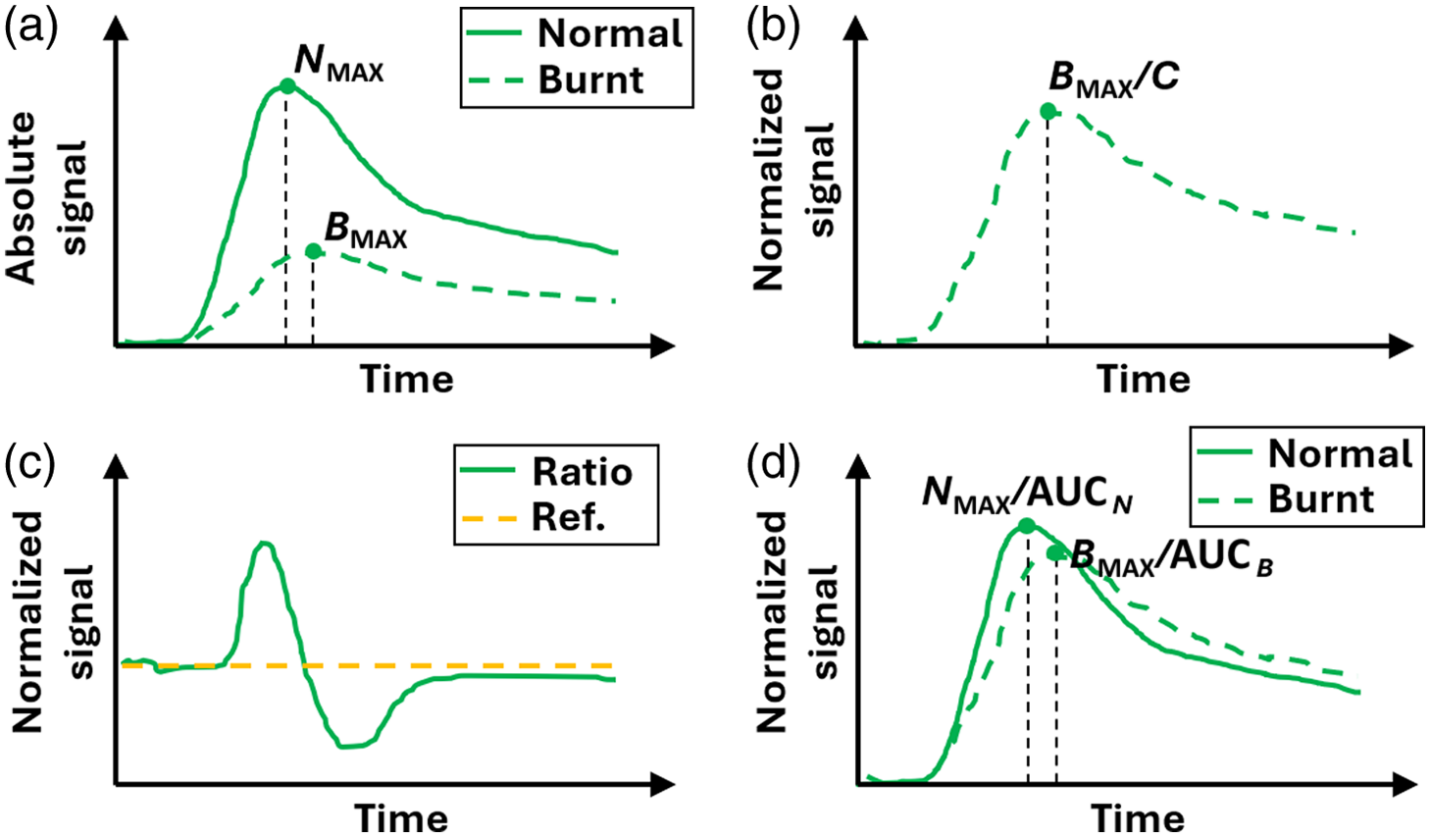
(a) Absolute burn (dashed line) and normal (continuous line) tissue signals. (b) Normalization by a constant. (c) SBR or CBR. (d) Normalization by AUC.

Normalization refers to the process in which the unknown signal coming from the injured tissue is compared with a reference signal coming from normal tissue.^18^^,^^24^ As in the previous steps of this pipeline, many different approaches exist which we will discuss below. Ultimately, the user must choose one according to their study type and goals. Figure 6(a) shows two fluorescence signals (one from the normal tissue, N(t), and another from the burn tissue, B(t)) before any normalization is performed. The most simple and immediate type of normalization is to generate a constant or scalar from N(t) to rescale B(t), which could be, for instance, the maximum value $N_{\mathrm{MAX}}$ of N(t), or its average value ⟨N(t)⟩. The outcome of this procedure is the signal B(t) without any change in its shape or timings, but only on its vertical extent. This is shown in Fig. 6(b), with the parameter C symbolizing any of the constants generated from the normal tissue signal. As shown in Sec. 5, this has the same effect as not normalizing at all, with the additional drawback that the timing information from the normal tissue signal is lost.

A second approach is to divide B(t) by N(t) [which can be called “signal-to-background ratio” (SBR)^27^], generating a signal such as the one in Fig. 6(c), so that the timing information from N(t) is retained and can be used for a time-wise comparison with B(t). If the two curves were very similar (or even identical), the result would be a horizontal line centered around 1 [orange line in Fig. 6(c)], whereas any differences will show up as deviations from this reference line. The main disadvantage of this approach is that the interpretation of the signal is not as straightforward as in the case of the absolute signal (in terms of identifying the value and position of maxima, inflow and outflow starts, inflow and outflow velocities, etc.).

Another strategy generating a signal qualitatively similar to that of Fig. 6(c) is the “contrast-to-noise ratio” (CBR),^39^ given by

$$\mathrm{CBR}(t)=\frac{B(t)-N(t)}{\mathrm{std}[N(t)]},$$ (4)

being std[N(t)] the standard deviation of the normal tissue signal. The main difference with respect to the SBR is that the reference value (horizontal orange line) is 0 and not 1, but the same disadvantage is shared.

Finally, we include a comparison between the burn and the normal signals in which no mathematical operation among them is involved; instead, each of them is normalized by its own area under the curve (AUC) (${\mathrm{AUC}}_{N}$ for the normal tissue signal and ${\mathrm{AUC}}_{B}$ for the burnt tissue signal), as shown in Fig. 6(d). Then, different features from each curve can be extracted and directly compared with each other. The main advantage of doing this is that, if correctly performed, it can properly account for differences arising from intersubject variability, instrumentation, ICG injection, and so on. This will be further discussed in Sec. 5.

### 3.8 Features Extraction (H)

**Fig. 7:**
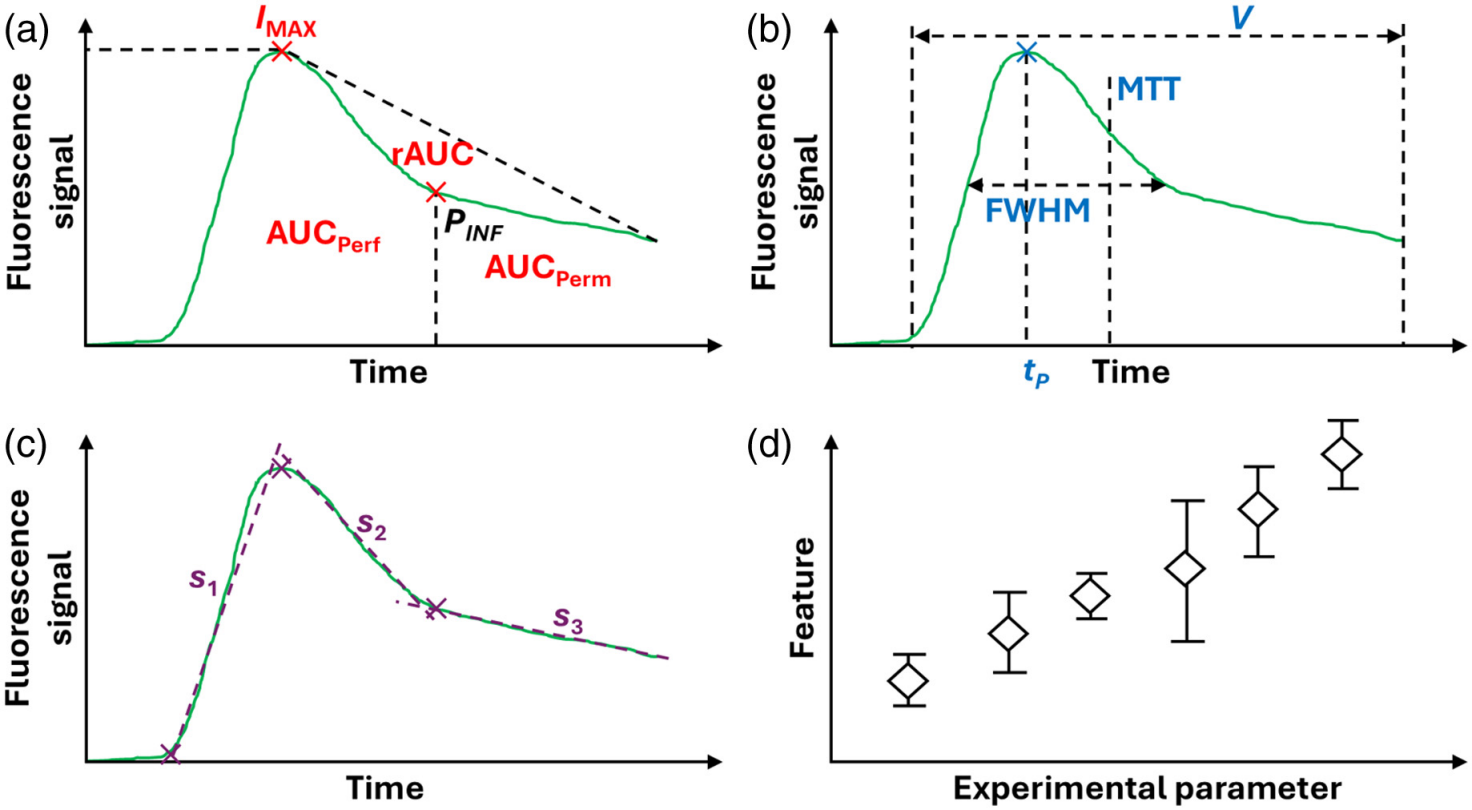
(a) Intensity features. (b) Timing features. (c) Mixed features. (d) Example of plot showing any of the previous features versus an independent experimental parameter of interest.

At this point, it is time to extract information from the fluorescence signal. This information can be thought of as features that characterize the kinetics curves. There are several different types of features, and it can be argued that their combination will not be the same for a signal coming from healthy tissue compared with a signal coming from burn tissue. In this work, we propose classifying the features into three different categories. Intensity features [Fig. 7(a)] strongly depend on the intensity of the signal detected by the camera without regarding the times at which these features occur. Examples of this are the peak value ($I_{\mathrm{MAX}}$), the area under the curve between the start point and the inflection point $P_{\mathrm{INF}}$ (associated with perfusion and denoted as ${\mathrm{AUC}}_{\mathrm{Perf}}$), and the area under the curve between $P_{\mathrm{INF}}$ and the end of the acquisition (associated with permeability and denoted as ${\mathrm{AUC}}_{\mathrm{Perm}}$).^35^ The shape of the decay from the peak value to the end of the curve (i.e., the last data point acquired), which in general is very distinctive between healthy and damaged tissues, can be used to define a residual area under the curve (rAUC).

The second kind of feature is the timing features [Fig. 7(b)], which are highly linked with the time at which identifiable events take place, but with little or no dependence on the corresponding intensity values. Among them, we can count the time to peak ($t_{P}$), the full width at half maximum (FWHM), the mean transit time (MTT), and the variance (V). The MTT can be computed as

$$\mathrm{MTT}=\frac{\overset{N}{\underset{i=1}{\sum }}F_{i}t_{i}}{\overset{N}{\underset{i=1}{\sum }}F_{i}},$$ (5)

where the index i refers to the time bin (so that $t_{1}$ and $t_{N}$ are the first and the last time bins, respectively), and $F_{i}$ is the detected fluorescence signal at time $t_{i}$, whereas V can be calculated as

$$V=\frac{\overset{N}{\underset{i=1}{\sum }}F_{i}t_{i}^{2}}{\overset{N}{\underset{i=1}{\sum }}F_{i}}-{\mathrm{MTT}}^{2}.$$ (6)

As a matter of fact, the quantity $\sum F_{i}$ in Eqs. (5) and (6) is none other than the area under the curve used for normalizing the kinetics curves, as defined at the end of Sec. 3.7.

Finally, the mixed features [Fig. 7(c)] depend both on the intensity values of the identifiable points and the times at which they show up in the curves. For instance, it is possible to fit the uptake section, the fast washout, and the slow washout with linear models to retrieve an ingress slope ($s_{1}$), a fast egress slope ($s_{2}$), and a residual egress slope ($s_{3}$).

This list of features only includes those that are fairly easy to identify, thus being far from exhaustive. Moreover, the features listed here can be combined to generate new features.

### 3.9 (Semi-)Quantitative Analysis (I)

Once the features have been chosen and extracted, they must be contrasted with independent experimental parameters to test for correlations. In the case of animal models, it is common to run ICGA experiments for burns created at different temperatures and times, so one can plot any of the features from the previous section against temperature and or time to look for trends [as schemed in Fig. 7(d)]. In the case of human patients and when biological samples are available, the independent experimental parameter could be the depth of burn estimated by histological studies. In addition, statistical tests should be performed to determine whether the dependence between features and parameters is significant or not.

The discussion provided in the previous paragraph is an example of a semi-quantitative analysis.^35^ A fully quantitative analysis requires explaining the features mentioned here in terms of biological parameters such as the blood, plasma, and interstitial volume fractions per volume of tissue; the permeability-surface area product; the extraction fraction; the ICG concentration; and flow rate, for which mathematical models (usually in the form of coupled differential equations) are needed.^27^^,^^35^^,^^40^

## 4 Materials and Methods

Here, we present the details of the swine study performed to test our proposed processing and analysis pipeline. As such, we include all the necessary information to maximize the reproducibility of this study.

### 4.1 Study Design and Animal Model

Two 3- to 4-month-old (∼70  kg) female market animal cross-breed pigs were obtained from the Swine Research and Teaching Center, a University of Wisconsin (UW) bio-secure farrow-to-finish swine facility. The pigs were individually housed in a humidity-controlled environment for 1 week of acclimatization prior to study commencement. On the first day of the study, animals were premedicated with intramuscular Telazol and xylazine for sedation, and ear venous access was obtained to receive a continuous infusion of lactated ringers. The pigs were weighed, transferred to the operating room, and placed in a ventral recumbency position. They were preoxygenated with 100% oxygen, intubated, and maintained on inhaled anesthesia with 1% to 5% isoflurane for the entirety of the procedure. The dorsal aspect of the animal was shaved and prepped with iodine. A stainless steel cylindrical burn device was electronically heated to a temperature of 150°C and was applied to the dorsal aspect of the pig for varying amounts of time to create circular burns with a 3.5-cm diameter spaced ∼3 to 5 cm apart. Dynarex (Bacitracin Zinc Oinment USP) and Cuticerin (Smith and Nephew) gauze dressing were applied to each wound followed by the application of gauze dressing and a tightly fitted shirt to prevent contamination of the wounds. The animal was awakened from anesthesia and returned to individual housing. Post-operative analgesia included buprenorphine, carprofen, and low-dose ketamine intravenous injection as needed. On post-burn day 2, the animal was premedicated, intubated, and anesthetized as described above. The dressings were removed, and the burns were cleaned with alcohol to remove any residual bacitracin. With the OnLume imaging device positioned 30 cm above the region of interest, the animal received a 7-mg intravenous injection of ICG, and angiography was performed (see Sec. 4.2). When the procedure was complete, wound care was performed as detailed previously, the animal was awakened from anesthesia and returned to its housing unit. This study was approved by the UW Institutional Animal Care and Use Committee.

### 4.2 Data Acquisition

**Fig. 8:**
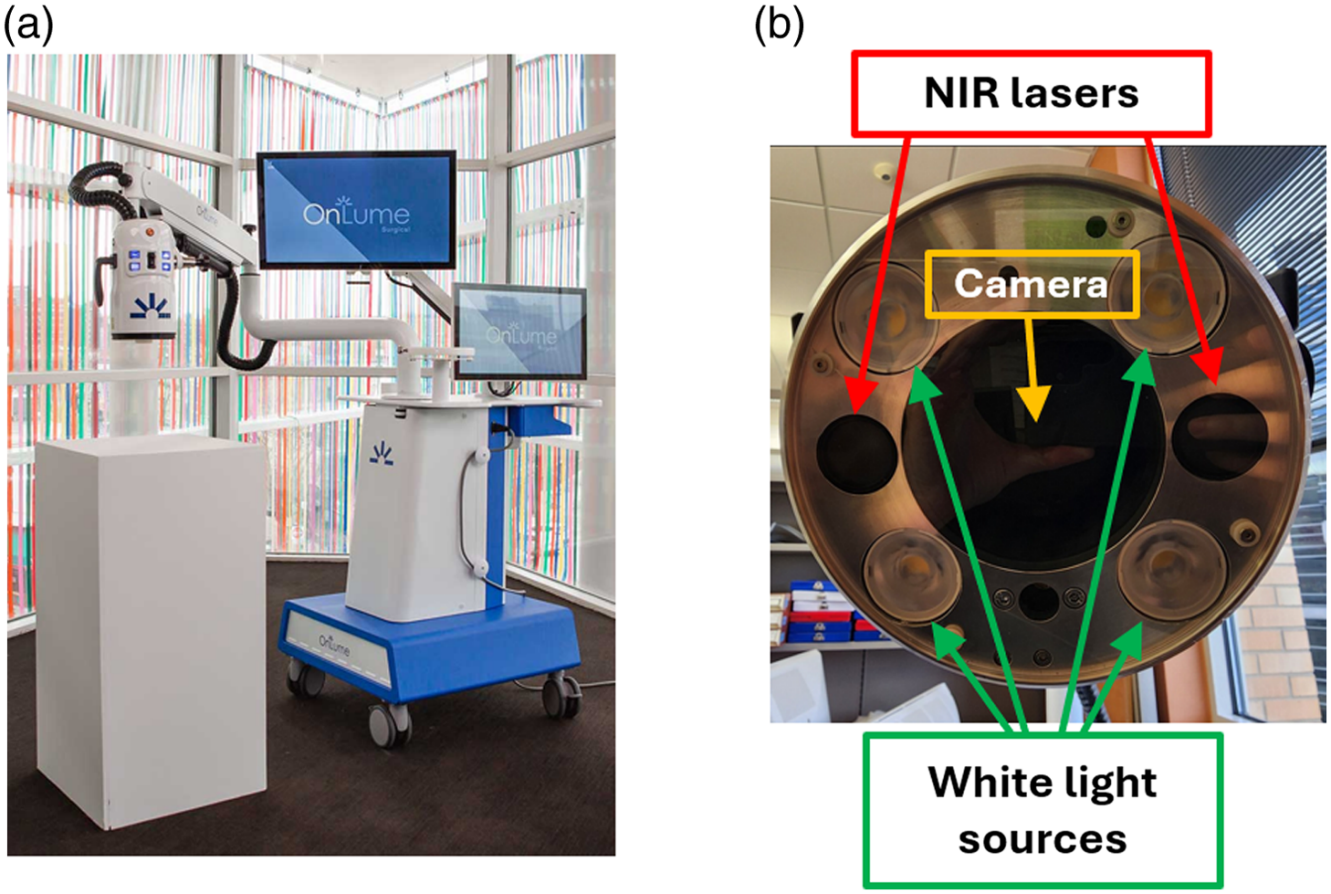
(a) Imaging system used in this study.^31^ (b) Bottom side of the imaging head depicting the position of the NIR laser sources, the white light sources, and the camera.

All the measurements were carried out with the help of an OnLume Avata Imaging System (OnLume Surgical Inc., Madison, Wisconsin, United States), shown in Fig. 8(a). Figure 8(b) presents the bottom side of the imaging head: two laser sources with beam spreading optics (805 nm, FWHM< 3 nm, power density of $23\;\mathrm{mW}/{\mathrm{cm}}^{2}$ at a WD of 30 cm) located on the opposite sides of the imaging camera illuminate the target tissue exciting the ICG molecules previously injected into the bloodstream. The video acquisition (frame size of 2048×1536  pixels, native framerate of 15 fps, and focal length between 16 and 85 mm) started at the time of ICG injection and continued for ∼2  min. The imaging device is capable of recording fluorescence video (filtered through a dichroic mirror at 801 nm, a long-pass filter with 808 nm cut-on, and a colored glass filter with 830 nm cut-on) as well as white light video simultaneously, enabled by four additional white light sources placed on the sides of the camera [Fig. 8(b)]. The white lights and excitation lasers are temporally multiplexed and synchronized with the image capture at a high enough frequency (180 Hz) that the white light appears steady. In addition, the white light illumination and capture operate at a duty cycle of less than 10%, with laser illumination and fluorescence capture operating at over 90% duty cycle to maximize the captured fluorescence signal. The software then averages over 12 captured frames for each display frame resulting in a 15-fps display by default and can be configured to operate at a 30-fps display by averaging six captured frames through software settings. The acquisition parameters can be controlled from a 21.5-in. touchscreen, whereas another 27-in. screen can be used to track the evolution of the measurement session (fluorescence, white light, and overlay fields displayed simultaneously) far away from the control touchscreen.

### 4.3 Data Processing and Analysis

Data processing and analysis followed the pipeline introduced in Sec. 3; to this end, MATLAB R2023b (MathWorks, Natick, Massachusetts, United States) was used. All the mandatory steps described in Sec. 3 were applied, whereas different combinations of the optional steps were chosen, giving as a result four specific pipelines (Table 1). Then, the outputs from each of these pipelines were compared.

**Table 1:** List of specific pipelines used to process the data from the swine study. MAC was applied to each of these pipelines if found necessary.

Optional steps	Pipeline 1 (NP)	Pipeline 2 (nAUC)	Pipeline 3 (FFC)	Pipeline 4 (FFC + nAUC)
FFC	—	—	X	X
nAUC	—	X	—	X

NP, no (optional) processing; FFC, flat field correction; nAUC, normalization by AUC.

**Fig. 9:**
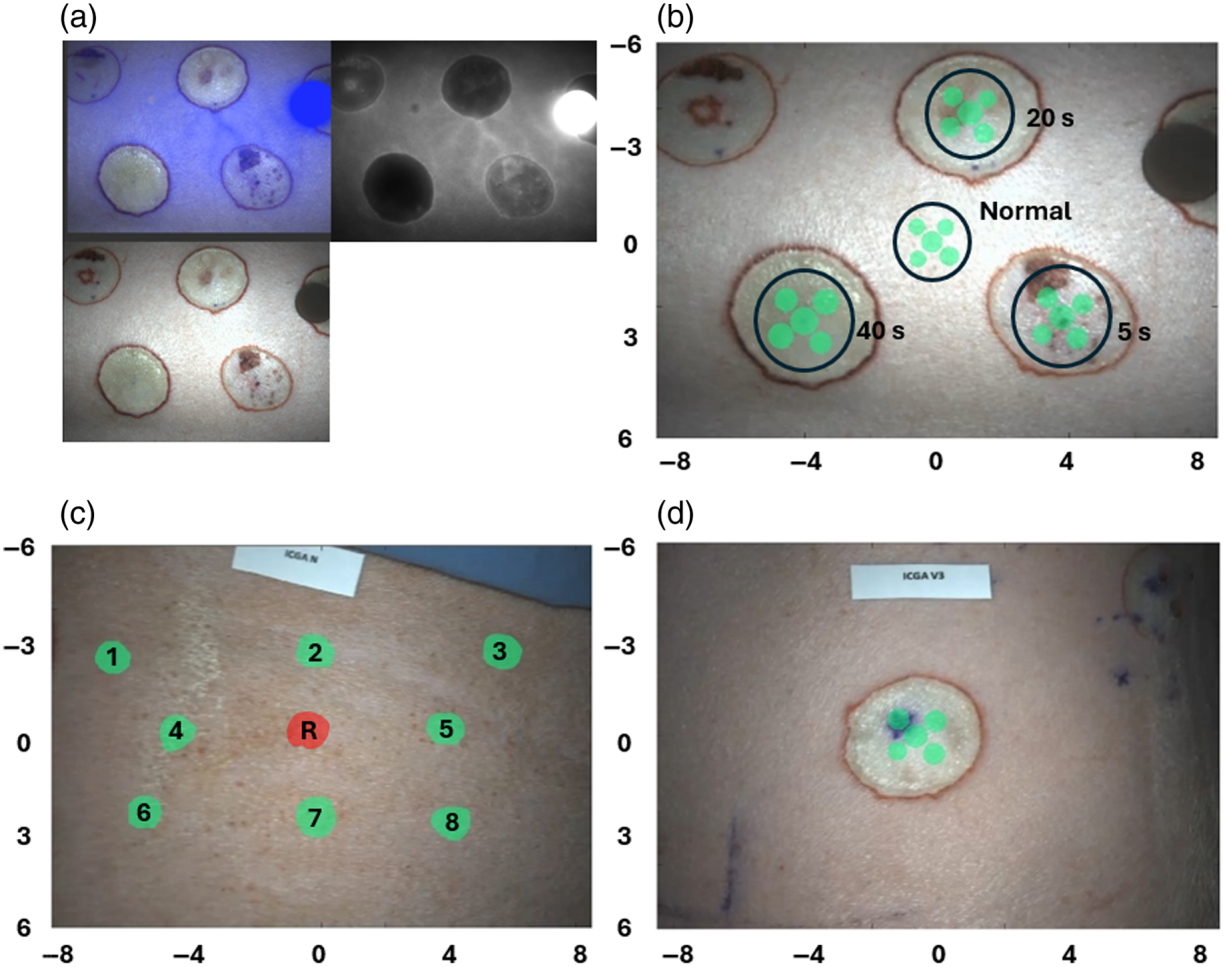
(a) White light field (bottom left), fluorescence field (top right), and overlay (top left) frame from the ICGA video for pig #1 (three-dimensional-printed standard target pictured in select cases). (b) Frame example of ICGA video for pig #1 including all ROIs. (c) ROIs from normal tissue study for pig #2, with the red central one used as reference (R). (d) Frame example of ICGA video for 20-s burn from pig #2.

Once the raw video files were loaded in the processing and analysis script (Fig. 9), they were split into a white field (WF) video and a fluorescence field (FF) video. The WF video was used to select the ROIs [Figs. 9(b)–9(d)] that were then collocated on the FF video to calculate each average ICG kinetics curve. Five circular ROIs with a 1-cm diameter were obtained from the center of each burn as well as from normal tissue regions. This size was selected to minimize the amount of heterogeneity within the ROI given that selecting the entire burn region, including the peripheral aspect, would include different kinetics phenomena that are lost during the averaging process. During this process, the frame rate was downsampled to 5 fps. The first pipeline in Table 1 is the most basic possible and is what can be found in other burn studies using ICGA.^6^^,^^18^^,^^20^^,^^22^ From there, the degree of complexity of the pipelines gradually increases, from a simple normalization by the area under the curve (pipeline 2) to a pipeline including a flat-field correction, motion artifact correction (MAC), and normalization by the area under the curve.

**Fig. 10:**
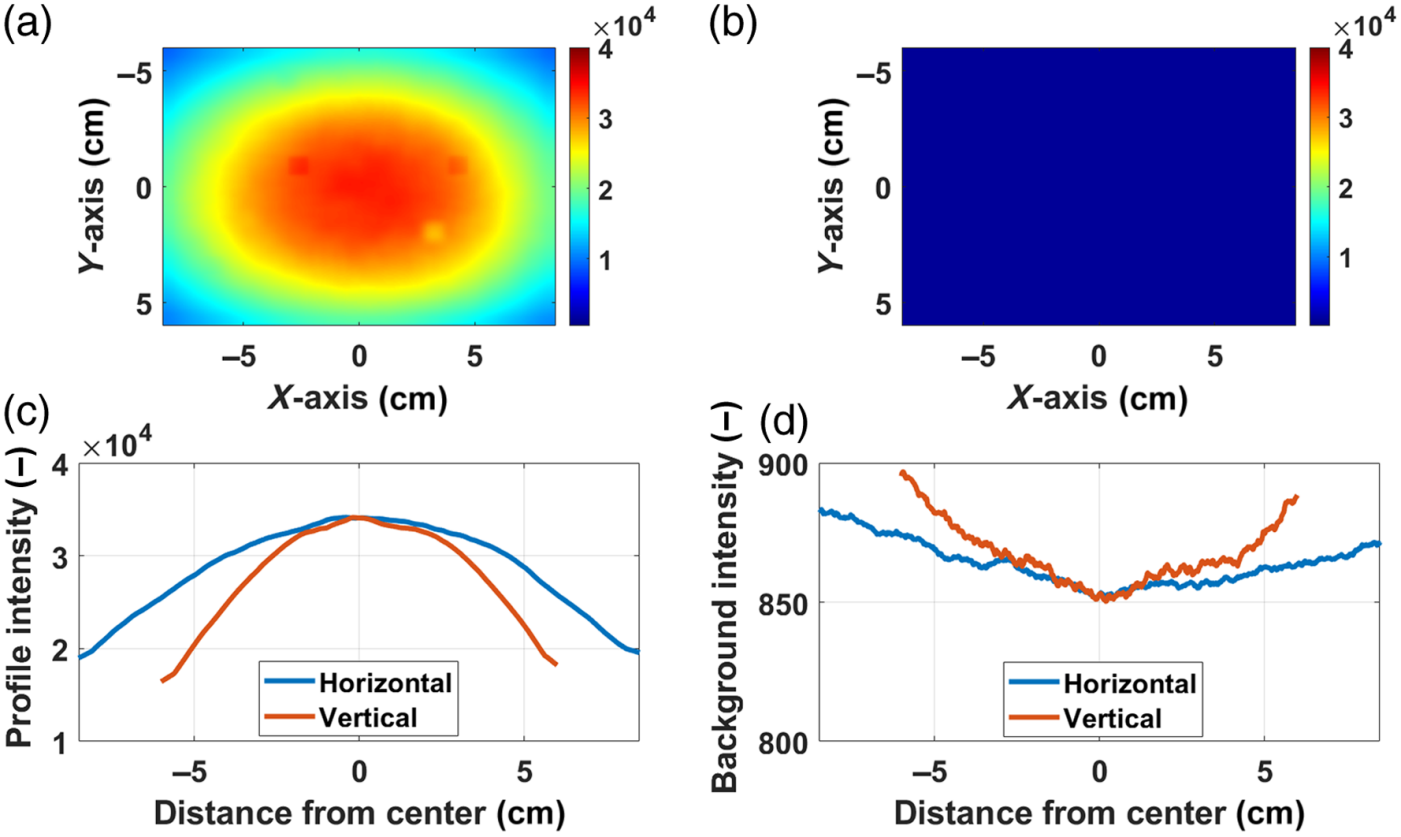
Flat field (a) and background (b) images used to apply the flat field correction. The corresponding horizontal and vertical profiles are shown in subplots (c) and (d), respectively.

The flat field image needed for the FFC described in Sec. 3.3 was acquired by imaging a liquid phantom with a mixture of deionized water, Intralipid (Sigma Aldrich, St. Louis, Missouri, United States), and India ink (Carbon Black Pigment, Speedball Art Products, Statesville, North Carolina, United States) to mimic typical tissue scattering and absorption properties, adding enough ICG to get a good signal-to-noise ratio but avoiding saturation [Fig. 10(a)]. The background image [Fig. 10(b)] was then subtracted from both the raw and the flat field images as shown in Eq. (1). The horizontal and vertical profiles [Figs. 10(c) and 10(d)] for the flat field and the background images, respectively, show that (i) the surfaces imaged with this device are not uniformly illuminated and (ii) the influence of the ambient light on the measurements is negligible.

**Fig. 11:**
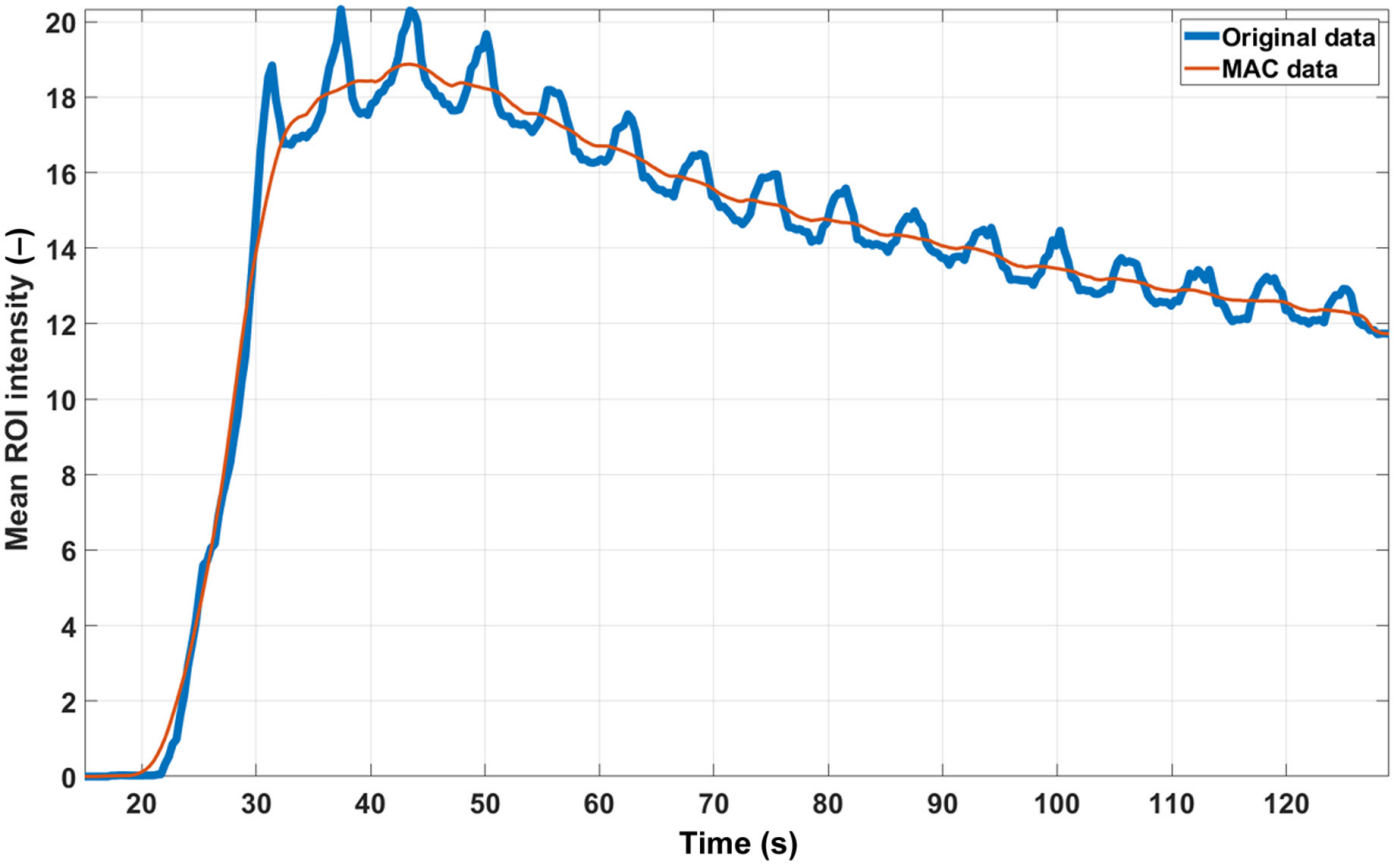
Example of ICG kinetics curve with motion artifacts (blue) and its motion artifacts-corrected version (orange) for pig #2.

Regarding the motion artifacts correction, some kinetics curves presented very regular and periodic spikes (likely due to the breathing rate), in which case simply smoothing the curves with a moving average window was enough (Fig. 11). Subtraction of the fluorescence signal from the initial baseline level was performed for every ROI; this was particularly necessary in the case of pig #2, which underwent several subsequent ICGA sessions with pauses of 15 min in between, meaning that the second up to the last video recording was characterized by an initial non-zero baseline signal level. No curvature or depth correction was used in this study. The reason for this is that the distance to the camera was kept constant for all the measurement sessions (as stated in Sec. 4.2), and also that the imaged surfaces were fairly flat.

Once the processing steps were fulfilled, six different features—two intensity, two timing, and two mixed features—were extracted from each ICG curve: the intensity features were the peak value ($I_{\mathrm{MAX}}$) and the rAUC, the timing features were the MTT and the FWHM, and the mixed features were the ingress ($s_{1}$) and egress ($s_{2}$) slopes. Although many more features can be extracted, the interpretation of the results gets largely complicated as this number rises. The reason for choosing the rAUC instead of the more intuitive ${\mathrm{AUC}}_{\mathrm{Perf}}$ or the total AUC is because of its behavior with the different normalization strategies tested here (particularly when normalizing with respect to the area under the curve of each kinetics curve). In addition, the MTT was favored against the time to peak due to its robustness against motion artifacts (especially for those pipelines in which no MAC was applied).

Next, we divided the study into two parts: first, we analyzed the behavior of the selected features when the pipelines were solely applied to normal tissue [Fig. 9(c)]. As in this case the only differences among the kinetics curves were expected to be explained just by the intrinsic heterogeneity of healthy tissue, the purpose of this part of the study was to define which of the specific pipelines minimized the variability introduced by instrumentation. Then, the second part of the study focused on comparing the extracted features for the different burns. Individual statistical analyses were performed for pig #1 (normal tissue, 5-s burn, 20-s burn, and 40-s burn) and pig #2 (normal tissue, 5-s burn, and 20-s burn) using the Mann–Whitney U-test, with a p-value <0.05 indicating statistical significance), followed by a group analysis (normal tissue, 5-s burn, and 20-s burn) applying the same statistical test.

Lastly, to simultaneously use all the parameters to conclude the severity of each burn, all the features were plotted using spiderweb graphs, and their geometrical properties were analyzed.

## 5 Results

### 5.1 Processing and Analysis Pipeline Applied to Normal Tissue

**Fig. 12:**
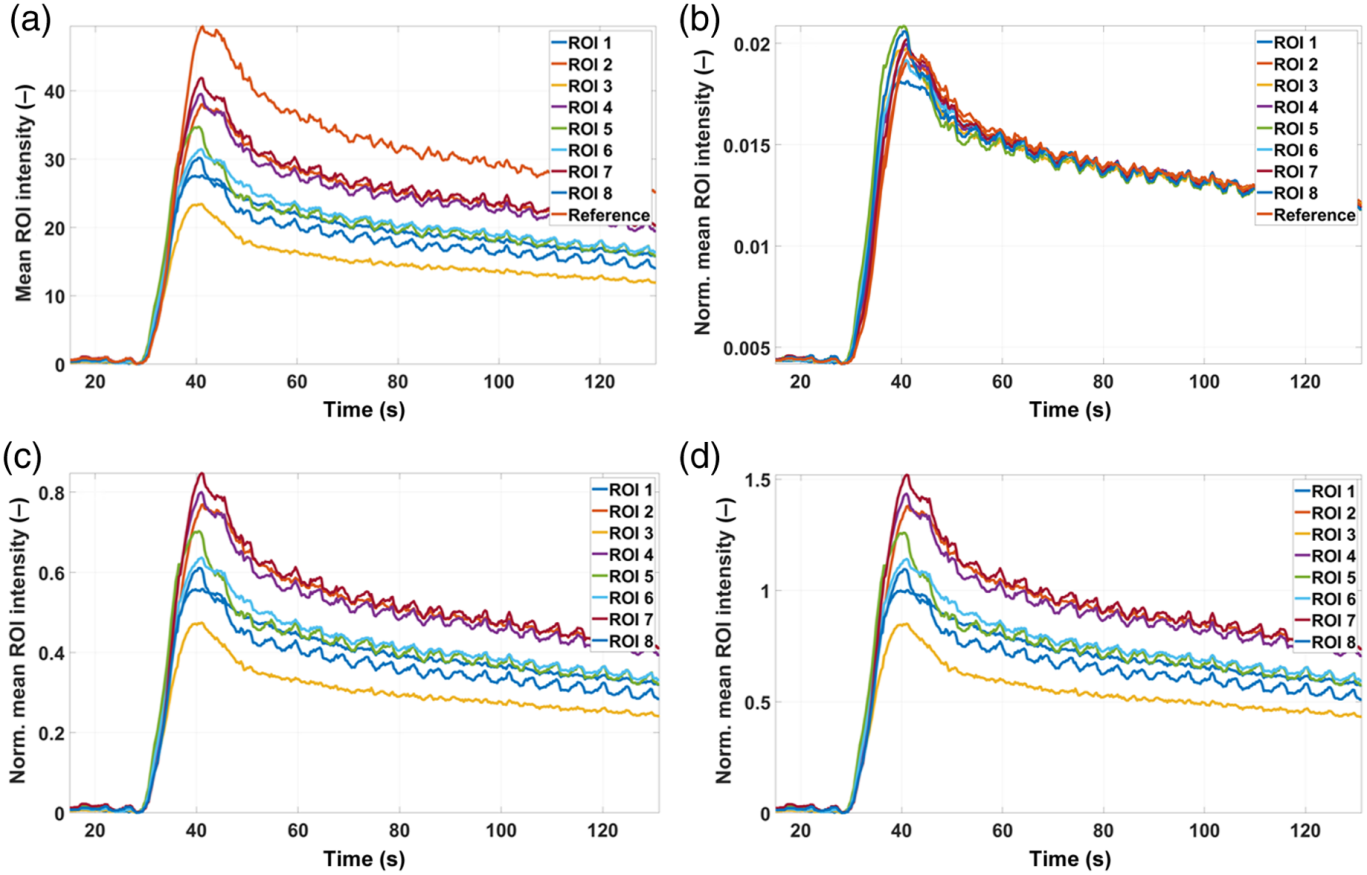
Comparison among average kinetics curves from the ROIs shown in Fig. 9(c) corresponding to normal tissue in pig #2. (a) No normalization. (b) Normalization by the area under the curve (nAUC) of each kinetics curve. (c) Normalization by the peak value of the normal tissue curve. (d) Normalization by the mean value of the normal tissue curve.

**Fig. 13:**
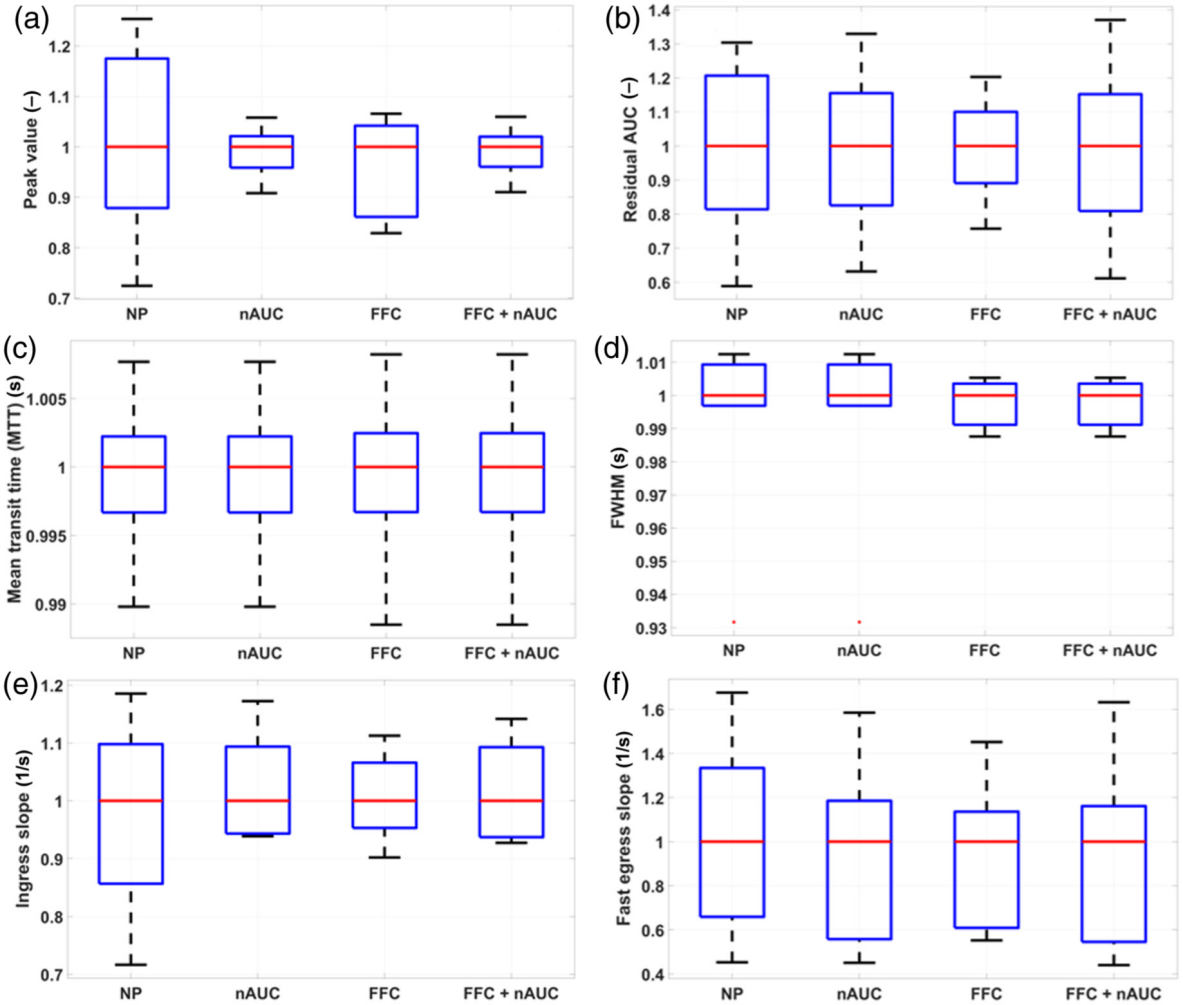
Boxplot comparison among relative features (i.e., features divided by their own corresponding medians) for different processing pipelines applied to ICG kinetics curves from normal tissue in pig #2. (a) Peak value. (b) rAUC. (c) MTT. (d) FWHM. (e) Ingress slope. (f) Egress slope. Red line: median; blue box edges: 25th and 75th percentiles; whiskers: minimum and maximum data points not considered outliers; red points: outliers. NP, no processing; nAUC, normalization by AUC; FFC, flat field correction.

Figure 12 shows a comparison of the ICG kinetics curves extracted from the ROIs depicted in Fig. 9(c) among different normalization strategies: (a) no normalization, (b) normalization by the AUC of each curve, (c) normalization by the peak value of the normal tissue curve, and (d) normalization by the mean value of the normal tissue curve. From Fig. 9(c), it can be seen that the most intense curve is the reference one corresponding to the central ROI, followed by the ROIs 2, 4, 7, and 5 and finally the ROIs 1, 3, 6, and 8; this distribution can be associated to the distance from each ROI to the center of FOV. In all cases, normalization rescales the curves without modifying their shape. In the case of normalizations (a), (c), and (d), the relative differences among curves’ shape remain the same because in each case, all the curves are divided by one and the same value; on the contrary, in (b), each curve is divided by its own AUC, giving as a result very similar ICG kinetics for all the ROIs (especially for the baseline, uptake, and residual washout), something expected if dealing with normal tissue. Again, the subtle differences (mainly around the peak) could be explained by the intrinsic heterogeneity of the tissue. In the following, no results for normalization with respect to normal tissue are shown because, essentially, this has the same overall impact on the curves as not normalizing at all. Finally, MAC was applied to those kinetics curves where the most important markers (the start of the uptake, the peak value, and the inflection point between the fast and the residual decays) were not easily identifiable. Next, Fig. 13 presents boxplot comparisons for the relative features (that is, each boxplot from each feature divided by the median of that feature) among different processing approaches. This is necessary to analyze how the variability of the features is affected by the processing strategy adopted; as stated before, a very small amount of variability is expected if dealing with normal tissue. In the case of the intensity and mixed features [Figs. 13(a), 13(c), 13(e), and 13(f)], the worst performance is obtained when no processing at all is applied. On the other hand, using the normalization by the area under the curve (nAUC) reduces the variability of the peak value and the ingress slope, whereas the FFC has its strongest impact on the rAUC [Fig. 13(b)]. The variability of the timing features [Figs. 13(c) and 13(d)] shows almost no dependence on the processing strategy adopted, which makes these very robust parameters to be used for intersession or intersubject studies. Finally, combining nAUC and FFC does not improve the variability of the data when compared with only using nAUC.

In conclusion, nAUC and FFC as optional processing steps have similar performances for the features included here; however, in general, nAUC is preferred because it can account for non-uniformities in the illumination field of the device and for uneven tissue surfaces, variations in the working distance and differences in the ICG administration process.

### 5.2 Processing and Analysis Pipeline Applied to the Assessment of Burn Wounds

**Fig. 14:**
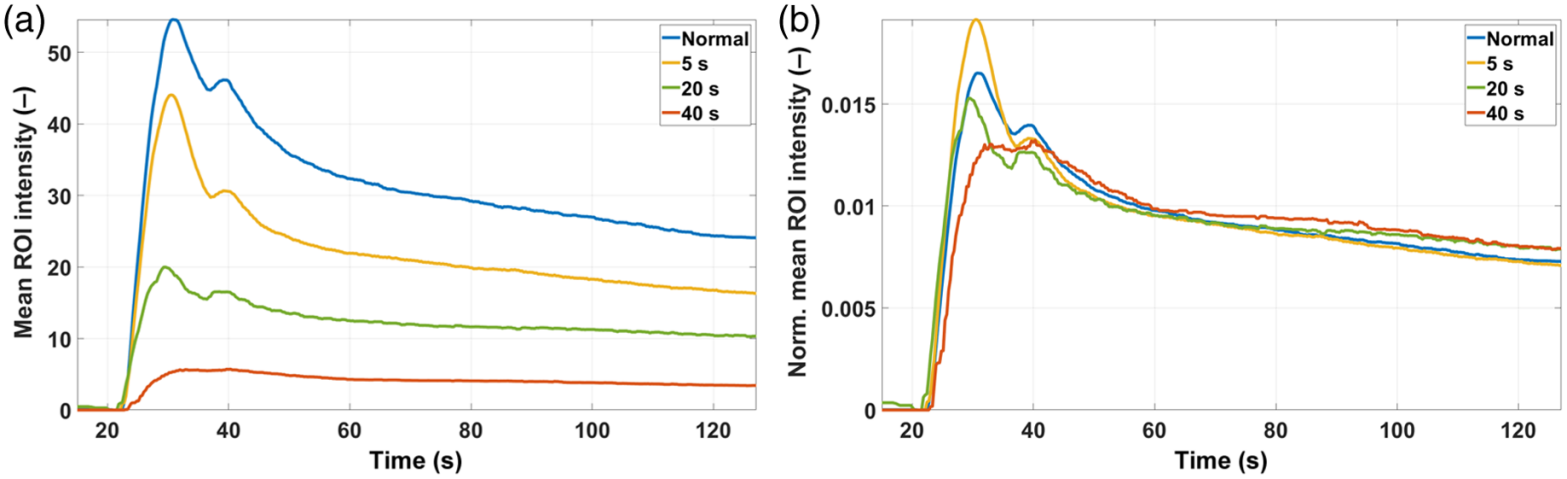
Representative mean kinetics curves for ROIs from pig #1 in Fig. 9(b). (a) Before nAUC. (b) After nAUC.

**Fig. 15:**
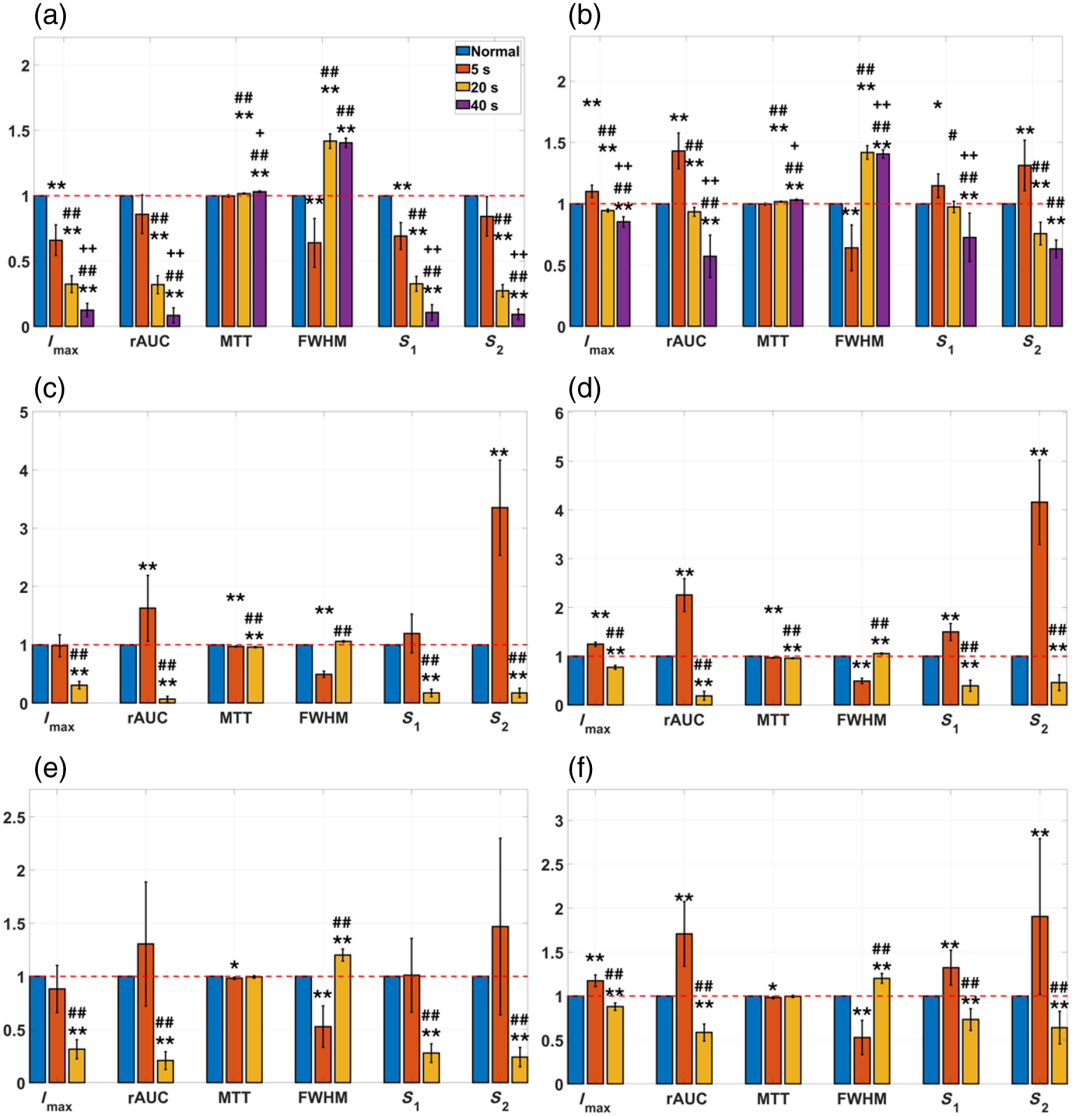
Bar plots for each feature (relative to the corresponding feature from normal tissue) for pig #1 without (a) and with (b) nAUC, for pig #2 without (c) and with (d) nAUC, and for the group without (e) and with (f) nAUC. The features are grouped by tissue type (normal, 5-s burn, 20-s burn, and 40-s burn). “*” indicates significant difference w.r.t. normal tissue; “#” indicates significant difference w.r.t. 5-s burn; “+” indicates significant difference w.r.t. 20-s burn; one symbol: p<0.05; two symbols: p<0.01.

As an example, a representative subset of the average kinetics curves for the ROIs from pig #1 before any normalization are plotted in Fig. 14(a), and the corresponding curves after nAUC are shown in Fig. 14(b). No MAC was needed in this case. Due to the presence of burn tissue, the variability of the curves is much larger than in the previous study with normal tissue. This variability is largely reduced by normalizing with respect to the area under the curve; as demonstrated before, in this way, the instrumental factors that increased the variability are mostly removed, whereas those arising from the different degrees of tissue damage remain (particularly in the range between 20 and 50 s), as it is visually evident from the features of each curve (from normal tissue to 40-s burn). Similar curves were obtained from pig #2 (not shown). A more quantitative analysis can be performed using bar charts as shown in Fig. 15—where each feature has been divided by the corresponding feature from the normal tissue—for pig #1 without (a) and with (b) nAUC, and for pig #2 without (c) and with (d) nAUC. In the case of pig #1, prior to normalization, the features already present statistically significant differences among tissue types (normal or 5-s, 20-s, and 40-s burns). However, the general trend for the intensity and the mixed features is always the same: the longer the time period during which the burn was created, the smaller the value of these features. In a more complex scenario, where no information is available about the burn depth or the time during which the burn was created, it would be impossible to separate superficial from deep burns just by analyzing these features. On the other hand, the timing parameters chosen here (MTT and FWHM) show a more distinct behavior: more superficial burns present MTT and FWHM values smaller than 1, whereas the opposite occurs for deeper burns. This clear differentiation extends to the other features when nAUC is applied: in that case, the intensity and mixed features show values larger than 1 for more superficial burns and smaller than 1 for deeper burns; in addition, some differences among burns become more statistically significant. This distinct behavior among the types of burn suggests the presence of hyperperfusion in the case of the more superficial burn, whereas the deeper 20- and 40-s burns have lower $I_{\mathrm{MAX}}$, ingress slopes, and egress slopes, potentially related to vascular destruction in these deeper injuries.

In pig #2, the impact of applying a nAUC does not have an effect as strong as in pig #1: it improves the differences between superficial and deep burns for $I_{\mathrm{MAX}}$, and it also increases the statistical significance of the differences for the ingress slope; apart from that, the trends of all the features with or without normalization are qualitatively the same. The reason for this is the fact that the ICGA videos were recorded with the target ROIs placed at the center of the camera’s FOV. This is clear evidence that this experimental setup can minimize undesired instrumental effects and irregularities from the tissue surface, with the benefit that it can help reduce data manipulation in later post-processing stages.

**Fig. 16:**
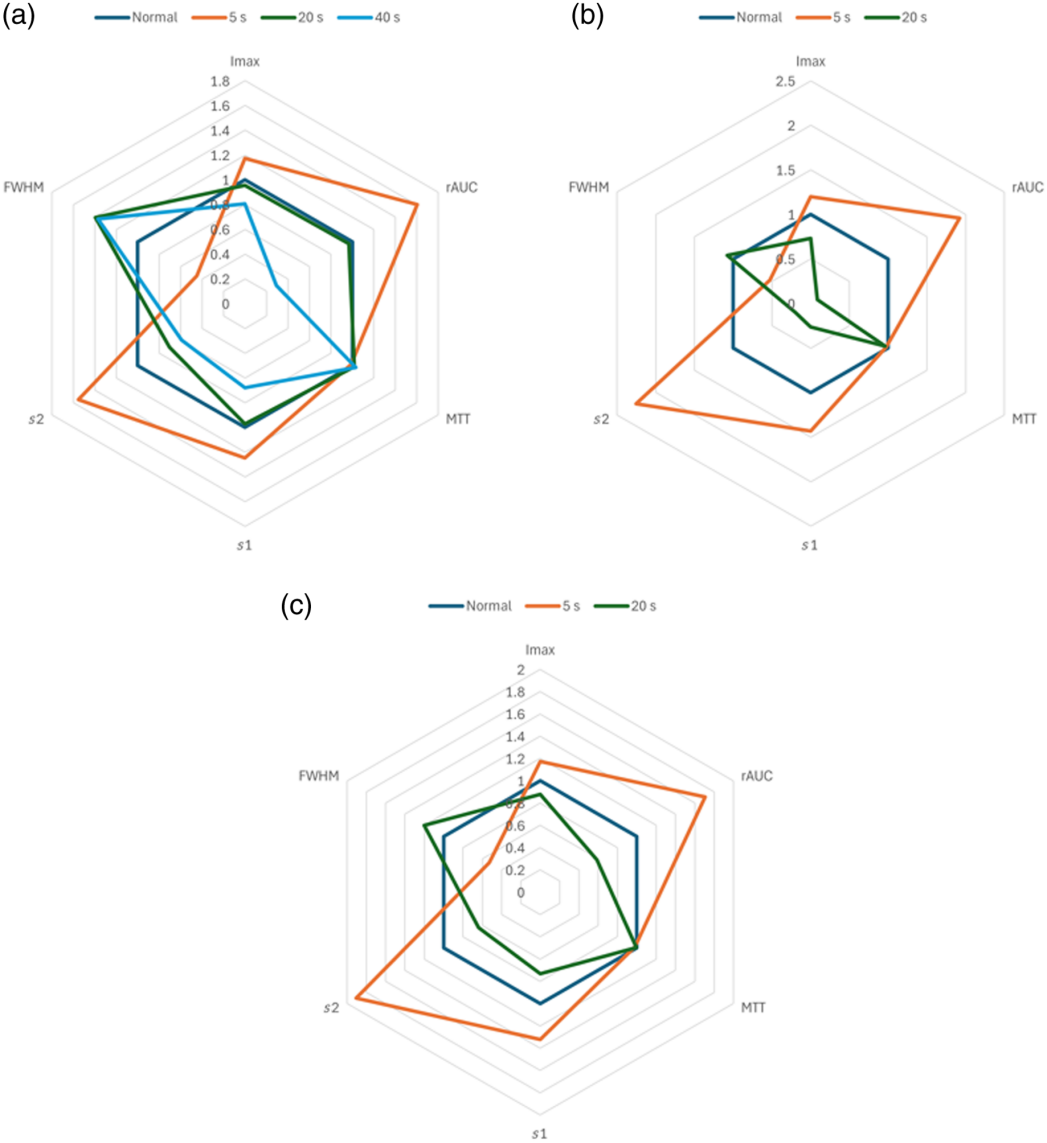
Spider charts for the features obtained from (a) pig #1, (b) pig #2, and (c) both pigs analyzed simultaneously, for normal tissue (blue), 5-s burn (orange), 20-s burn (green), and 40-s (cyan).

The group results are depicted in Fig. 15(e) (without normalization) and Fig. 15(f) (with nAUC). Here, the use of normalization by the area under the curve is more critical because it makes the differences among all tissue types more significant, and it particularly helps to discriminate between the more superficial burn at 5 s and the deeper one at 20 s. The previous analysis required considering each feature separately to decide on the burn depth. However, this could point to misleading conclusions. For instance, the behavior of the MTT between both pigs is different and hence confusing; the egress slope is the feature that suffers the largest change in pig #2, whereas in pig #1, the most sensitive features are the egress slope, the FWHM, and the rAUC. Therefore, a global analysis that accounts for all the features simultaneously would be much more useful. This can be done with the aid of spider charts. Figure 16 shows spider charts (generated with Excel v2501 for Microsoft 365) for the features obtained from pig #1 [Fig. 16(a)], pig #2 [Fig. 16(b)], and both pigs analyzed simultaneously [Fig. 16(c)] for each tissue type when nAUC is applied. The length of each spoke is proportional to the value of the feature it represents; once again, these features were divided by the corresponding ones from normal tissue as a reference. This is why the spider chart for the normal tissue looks like a perfect hexagon.

The way in which the features are ordered in these plots is crucial. Assuming these plots can be appropriately represented as ellipses,^41^ the orientation (defined as the angle with respect to an imaginary vertical axis and ranging from −90 to 90 deg) is positive for the 5-s burn and negative for the 20- and 40-s burns; as the spider chart for normal tissue can be thought of as a near-perfect circle, its orientation can be set to 0 deg to better separate between the two types of burns. According to this, in the case of pig #1, the spider chart orientations for the 5-, 20-, and 50-s burns are +35, −38, and −32  deg, respectively, whereas in the case of pig #2, the orientations are +35  deg for the 5-s burn and −34  deg for the 20-s burn. Last, the spider charts arising from the group also produce very distinctive orientations (+34 and −36  deg for the 5- and 20-s burns, respectively).

## 6 Discussion

In this work, we introduced a detailed processing and analysis pipeline applicable to ICGA data from burn studies that can be used to assess the severity of indeterminate burn wounds in an objective way. The only steps involving the user criteria are the selection of (i) the burn and reference normal tissue ROIs, (ii) the start time of the ICG kinetics curve, (iii) the need for motion artifacts correction, and (iv) the features to be extracted from them to perform the statistical analysis. As the focus of this paper is to describe the protocol for obtaining, processing, and analyzing ICGA data for clinical use, no histopathological studies were performed. Future work will explore the relationship between ICGA parameters and clinical features such as visual evaluation, healing potential, and histologic burn depth.

We applied this pipeline to a swine burn model in two pigs, each of them measured under different experimental conditions. Both datasets were processed using the same mandatory steps suggested in our pipeline, but with different combinations of the optional steps to compare the outcomes.

First, we demonstrated that some minimum level of post-imaging pre-processing is needed to correctly interpret the data to avoid unreliable variability of the ICG curves extracted from different ROIs pertaining only to normal tissue. This is a point that is very commonly ignored in basic ICGA studies^6^^,^^18^^,^^20^^,^^22^ but is inherent to fluorescence imaging systems and the interpretation of their data. There is nearly always high lateral inhomogeneity in the image which is not appreciated by the casual user, and this variation in intensity across the field of view, or from changes in the position of the camera can have profound changes in the calculated intensities. This itself is the largest hazard of misinterpretation when using ICGA as a quantitative tool.

Second, we showed that simply normalizing the ICG kinetics curves from the burn tissue with respect to a constant arising from the non-burn tissue kinetics is not sufficient and is equivalent to not normalizing at all. Instead, normalizing each curve by its own area under the curve can globally account for several uncontrolled experimental conditions, such as a non-uniform illumination of the imaging device’s field of view, the curvature of the tissue being imaged and possible differences in the ICG administration, among others. Although each of these factors may be accounted for separately, this implies a larger number of “interactions” with the data, sometimes resulting in excessive corruption of the measured information. In addition, correcting for motion artifacts when necessary enhances the proper determination of features such as the peak value, the time to peak, and the rate of change of the fluorescence signal with time.

Third, we classified the features from the kinetics curves into intensity, timing, and mixed features and showed that timing features remain almost unaltered when different processing steps were applied. This is reasonable when considering that the moments at which those features show up in the fluorescence signal depend only on the ICG kinetics in the tissue, without regarding how intense the signal is at those times. This means that timing features can be particularly helpful when comparing different measurement sessions from the same subject and/or from different subjects. These will allow translation among various manufacturers’ cameras and can be independent of the clinical teams’ technique in imaging.

Fourth, we managed to provide conclusions on the burn severity for each pig analyzed separately as well as by performing a group analysis, based on a set of features arising from the kinetics curves and consisting of the peak value and the residual area under the curve (classified as intensity features), the mean transit time and the full width at half maximum (timing features), and the ingress and egress slopes (mixed features). The increased values of the intensity and mixed features for the 5-s burn with respect to the normal tissue, as well as the reduced FWHM, suggests that this burn is more superficial and that the surviving viable tissue is hyperperfused; the exactly opposite behavior is seen for the 20- and 40-s burns (this last one only for pig #1), indicating that those burns represent full thickness with little or no viable tissue.

Fifth, and based on the observed heterogeneous behavior of each feature (especially the MTT) among pigs when analyzed individually, a quantitative global analysis method was proposed here that combines all the features at the same time. This method consists of generating spiderweb charts from the studied features and retrieving their orientations relative to normal tissue values. When the order in which the features are placed around these plots is appropriately chosen, the orientations for the more superficial and for the deeper burns are significantly different.

Finally, it is worth mentioning the technique study used here between the two sets of pig data analyzed. For the first pig study, only one ICGA video was recorded with all the involved ROIs in different positions with respect to the center of the device’s FOV, whereas for the second pig study, each ROI was centered at the FOV. There are similarities between features for pig #2 with and without normalizing the corresponding kinetics curves by the area under the curve; however, this is not true for the data from pig #1. Thus, the technique of imaging the ROI in the center provides minimal need for data post-processing steps; however, this approach is likely unrealistic for routine clinical use because of the disadvantage that more videos need to be recorded and multiple ICG injections are required for each ROI. However, this method was undertaken to illustrate the fact that the field of view of the ICGA imaging can affect the data extracted from the kinetic curves.

The main limitation of the pipeline described in this work is the fact that it strongly relies on the use of data measured from normal tissue. Although this can in principle be readily available in controlled studies based on animal models, in general, it is not possible to ensure access to normal tissue in the case of human patients. One option is to visually identify tissue regions close to the affected area being imaged that looked as normal as possible and then refer the measured data from different ROIs to that first one; this would minimize the number of acquisition sessions, but the proximity of the tissue identified as “normal” to the burned area may have altered kinetics due to surrounding inflammation in non-injured tissue. Another option is to measure ICG kinetics curves from healthy tissue in body parts distant from the affected region; however, this increases the number of measurement sessions; contralateral uninjured skin is ideal, but if this is unavailable, it should be recognized that perfusion and permeability properties of tissue differ across tissues and organs.^42^ If normal tissue is unavailable, population-based reference curves can be used.^42^ Undoubtedly, further work is necessary to investigate modifications to remove or at least mitigate this constraint.

Though the pig model provides valuable preclinical insights, human burn wounds exhibit greater variability in perfusion, thickness, and healing response. Our preliminary work in human subjects has revealed extensive interpatient variability in ICGA kinetics that is likely in part due to factors such as cardiac output, vascular disease, and inflammation. In fact, our studies in pigs were initiated in response to this interindividual variability, and the swine model studies provide confirmation about the challenges associated with ICGA imaging, prompting this paper. For further rigor, we will plan to analyze ICGA kinetics within a burn wound within a patient to evaluate visually different areas. We also anticipate the need for more robust strategies to correct for patient motion given this injection will be delivered while the patient is awake.

## 7 Conclusion

In summary, the processing and analysis pipeline proposed here demonstrated the need for a minimum amount of raw data processing to improve comparisons within and between studies. It also introduced a normalization by the area under the curve of the ICGA kinetics curves, in which case most of the uncontrolled experimental factors are removed from the study. In addition, a classification for the features arising from ICGA kinetics curves has been provided, and in particular, the so-called timing features proved to be almost independent of the processing steps included in the pipeline, implying that these features can be further used for robust translation between devices and teams studies. Moreover, a groupwise analysis of the features provided a clear way of separating deep burns from more superficial ones.

This study provides a way to enhance the robust reliability and accuracy of the ICGA measurements to assess the severity of indeterminate burn wounds. These conclusions need to be confirmed in human studies, followed by a level of automation of this processing pipeline to be incorporated in future instrumentation software to facilitate clinical studies and eventual adoption.

## Data Availability

The data that support the findings of this article are not publicly available but can be upon reasonable request to the corresponding author (gibson@surgery.wisc.edu).
